# Characterization of the *Populus Rab* family genes and the function of *PtRabE1b* in salt tolerance

**DOI:** 10.1186/s12870-018-1342-1

**Published:** 2018-06-18

**Authors:** Jin Zhang, Yu Li, Bobin Liu, Lijuan Wang, Li Zhang, Jianjun Hu, Jun Chen, Huanquan Zheng, Mengzhu Lu

**Affiliations:** 1 State Key Laboratory of Tree Genetics and Breeding, Key Laboratory of Tree Breeding and Cultivation of the State Forestry Administration, Research Institute of Forestry, Chinese Academy of Forestry, Beijing, China; 2 Biosciences Division, Oak Ridge National Laboratory, Oak Ridge, TN USA; 3 Developmental Biology Research Initiatives, Biology Department, McGill University, Montreal, Quebec, Canada

**Keywords:** *Populus*, Rab GTPase, Phylogenetic analysis, Co-expression network, Subcellular localization, Salt tolerance

## Abstract

**Background:**

Rab proteins form the largest family of the Ras superfamily of small GTP-binding proteins and regulate intracellular trafficking pathways. However, the function of the Rab proteins in woody species is still an open question.

**Results:**

Here, a total of 67 *PtRabs* were identified in *Populus trichocarpa* and categorized into eight subfamilies (RabA-RabH). Based on their chromosomal distribution and duplication blocks in the *Populus* genome, a total of 27 *PtRab* paralogous pairs were identified and all of them were generated by whole-genome duplication events. Combined the expression correlation and duplication date, the *PtRab* paralogous pairs that still keeping highly similar expression patterns were generated around the latest large-scale duplication (~ 13 MYA). The *cis*-elements and co-expression network of unique expanded *PtRabs* suggest their potential roles in poplar development and environmental responses. Subcellular localization of PtRabs from each subfamily indicates each subfamily shows a localization pattern similar to what is revealed in *Arabidopsis* but RabC shows a localization different from their counterparts. Furthermore, we characterized *PtRabE1b* by overexpressing its constitutively active mutant PtRabE1b(Q74L) in poplar and found that PtRabE1b(Q74L) enhanced the salt tolerance.

**Conclusions:**

These findings provide new insights into the functional divergence of *PtRabs* and resources for genetic engineering resistant breeding in tree species.

**Electronic supplementary material:**

The online version of this article (10.1186/s12870-018-1342-1) contains supplementary material, which is available to authorized users.

## Background

Plant cells are divided into several biochemically distinct membrane-bound organelles, which have specific functions permitting the maintenance of specialized environments for various chemical reactions [1]. Communication and transport between these membrane compartments are vital to not only basic cellular activities but also development and environmental responses. This transport is maintained through complex and precise regulation pathways, which includes membrane fusion between transport vesicles and target organelles [2]. The vesicular fusion is mediated by two important group proteins: the soluble N-ethylmaleimide-sensitive factor attachment receptors (SNARE) and the Rab GTPases [1].

Rab GTPase are involved in the entire process of vesicle transport including budding from donor organelle, docking, tethering and fusing with the target membrane [3, 4]. Similar with other small GTPases, Rabs can be recycled between the GTP-bound active form and GDP-bound form; and this recycle mechanism is conserved in all eukaryotes [5]. There are four conserved motifs involved in nucleotide binding and hydrolysis in Rab proteins. Mutating the specific residues can generate dominant negative or constitutively active forms that exhibit altered nucleotide-binding or hydrolysis characteristics, which can be used to investigate Rab functions [1].

The Rab family in plants is divided into eight subfamilies (RabA-RabH) [6]. Each type of Rab has a characteristic distribution on organelle membranes. In other word, each organelle has at least one type of Rab proteins on its cytosolic surface [7]. *Arabidopsis* RabA1 GTPases are involved in transport between the *trans*-Golgi network (TGN) and the plasma membrane (PM) [8]. Tobacco NtRab2 (RabB) regulates vesicle trafficking between the endoplasmic reticulum (ER) and the Golgi bodies [9]. RabD is involved in ER–Golgi traffic [10]. RabE has the Golgi apparatus and PM localization [11, 12]. RabF/Rab5 and RabG/Rab7 are major regulators of endosomal/vacuolar trafficking in eukaryotes. RabH is located in Golgi apparatus [13]. The hypervariable C-terminal domain (HVD) of Rab proteins is post-translationally modified by isoprenyl moieties, which is important for protein target to membrane structure. But the complete mechanism is needed to elucidate the localization [14–16].

As the key player in fundamentally cellular activities, plant Rab proteins are involved in various regulatory processes of development and stress response. For example, Rab proteins are implicated in pollen tubes growth [9], leaf morphology [12], xylem development [17], autophagy [18], salinity stress [19] and pathogen defense [12]. Eukaryotic cells recognize their environment mainly through proteins on the PM, including receptors and sensors, which evoke intracellular signal transduction to respond to environmental cues. To date, membrane traffic and associated members are well studied in yeast, mammalian and *Arabidopsis* cells. However, there were finite studies on Rab GTPase functional characterization in woody plants. Different with the annual plants, perennial woody species not only undergo seasonally environmental changes, but also have specific developmental processes such as strong secondary growth. Therefore, study the *Rab* gene family is important to understand the regulation of trafficking during these specific environmental response or development in woody plants. As perennial woody model plant, poplars are widespread distributed group of economic species having the rapid growth and high production of plant biomass features [20]. The expansion of gene family along with the whole genome duplication events of *Populus* genome also provide the possibility for functional divergence of genes.

In this study, we comprehensively analyzed the *Populus Rab GTPase* gene family. A total of 67 *PtRab* genes were identified and categorized into eight subfamilies in *P. trichocarpa*. And 27 paralogous pairs were identified and all of them were generated by whole genome duplication (WGD) events, which implying tandem duplication was missed in *PtRab* family expansion. The *PtRab* paralogous pairs with highly similar expression patterns were generated around the latest WGD event (~ 13 MYA). In addition, the different localization differences among *PtRab* subfamilies provide basis for their functional divergence. Based on the *cis*-acting elements and the co-expression network, several *Rab* genes were associated with stress response. Among them, *PtRabE1b* was significantly induced by salt stress. Finally, we overexpressed constitutively active mutant PtRabE1b(Q74L) in poplar and found PtRabE1b(Q74L) enhanced the salt tolerance in poplar. These findings provide new insights into the functional divergence of *PtRab* genes and resources for genetic engineering resistant breeding in tree species.

## Results

### Identification and phylogenetic analysis of *Rab* gene family in *Populus*

**Fig. 1:**
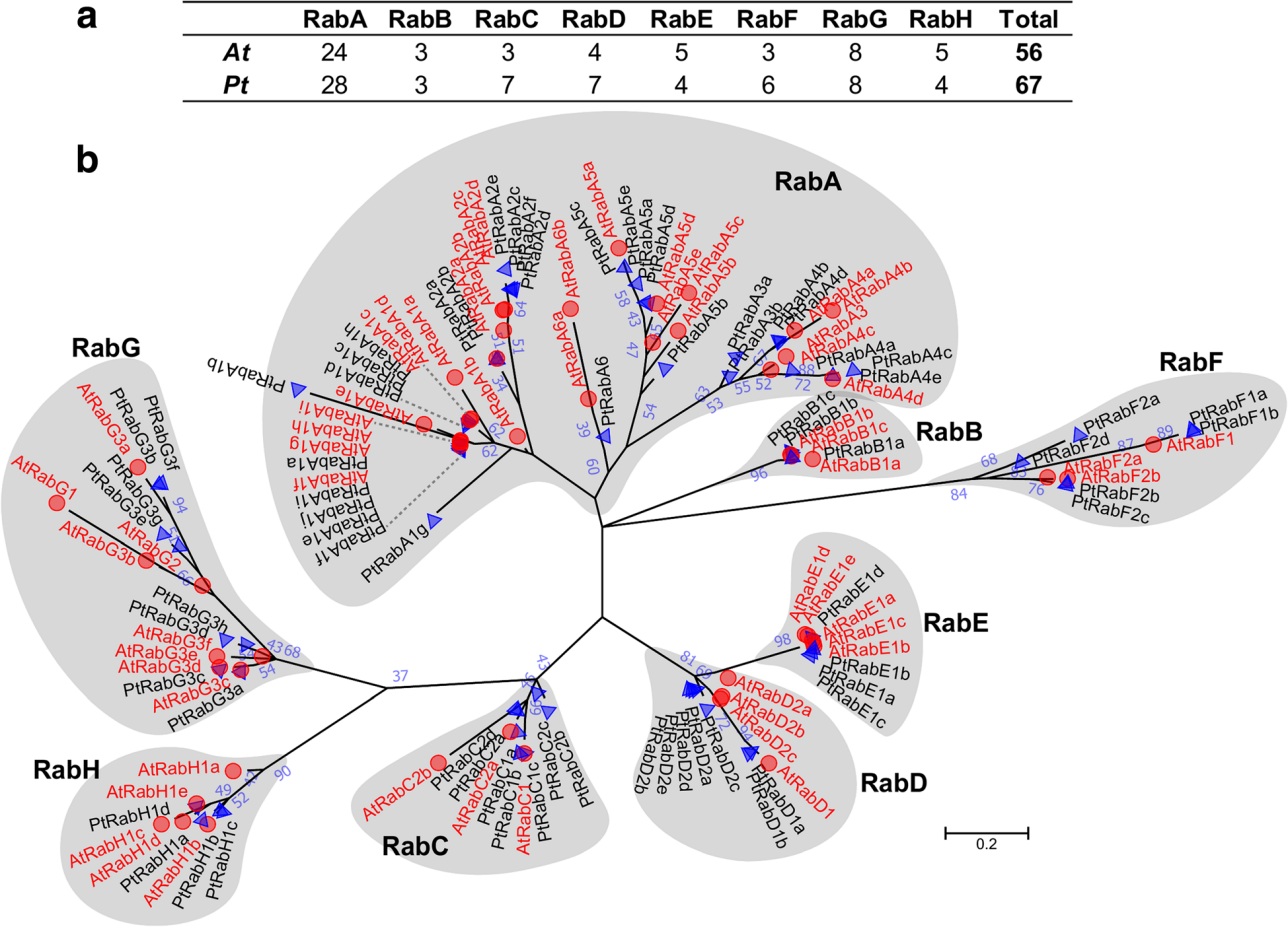
Rab family members and their phylogenetic relationships in *A. thaliana* and *P. trichocarpa*. **a** Eight Rab subfamilies (*RabA-H*) members in *A. thaliana* (*At*) and *P. trichocarpa* (*Pt*). **b** Phylogenetic tree was constructed using full-length protein sequences by the maximum likelihood (ML) method with 1000 bootstrap replicates using MEGA 7.0.26. Numbers above branches of the tree indicate percentage of bootstrap values

To identify *Rab GTPase* genes in *Populus*, the 56 known *Arabidopsis Rab* sequences [1] were used as queries in BLAST searches against the *P. trichocarpa* genome (release V3.0). A total of 67 putative *Rab GTPase* genes were identified in *Populus* (Additional file 1: Table S1). To examine the phylogenetic relationships of the *PtRab* gene family, a multiple sequence alignment was performed using full-length protein sequences of PtRabs and AtRabs, and a phylogenetic tree was constructed. As shown in Fig. 1, the *PtRab* family was grouped into eight clades (*RabA-RabH*) based on the sequence similarity and *PtRabs* were named according to their orthologous in *Arabidopsis.* Compared to *Arabidopsis*, four (*RabA*, *RabC*, *RabD* and *RabF*) of eight *Rab* subfamilies were extended in *Populus*. The *Populus RabB* and *RabG* subfamilies had the same size with *Arabidopsis*, while *Populus RabE* and *RabH* subfamilies were smaller than *Arabidopsis*.

### Structure of *Rab* genes and conserved motifs of Rab proteins in *Populus*

To further investigate the structural features of *Populus Rab* genes, their exon-intron structures and protein motif composition were analyzed. Structure divergence of exon-intron play a pivotal role during evolution. Generally, paralogous genes are highly conversed in gene structure and this conservation is sufficient to reveal their evolutionary relationships [21]. In *Populus*, *Rabs* within the same subfamily shared similar gene structures (intron number and exon length), especially the members in *RabA*, *RabC* and *RabH* subfamilies. Compared to other multi-intron *Rab* genes, the members in *RabA* subfamily only have one intron (Additional file 2: Figure S1). Despite the gene length and gene structures were varies among the members in different *Rab* subfamilies, the protein length of most Rabs was consistently ~ 200 aa in both *Populus* and *Arabidopsis*.

Classical Rab proteins including four conserved motifs, which were involved in nucleotide binding and hydrolysis. We then analyzed the conserved motifs in PtRabs. Like Rabs in many other species, almost all the PtRabs including the four conserved motifs (motif 1–4; Additional file 2: Figure S1B). Although a little sequence bias was existed in the four motifs among different Rab subfamilies, the basic framework of the four motifs was relatively conserved in PtRabs. Based on previous studies, several specific amino acids in the conserved motifs of Rabs are involved in nucleotide binding and hydrolysis [1, 11]. In the PtRab family, three residues (S in motif-1, Q in motif-2 and N in motif-3; asterisks in Additional file 3: Figure S2) could be used to generate the dominant negative or constitutively active mutants through altering nucleotide-binding or hydrolysis characteristics for functional investigation.

Restricted by currently available annotation of the *Populus* genome, the structures of several genes were not well characterized. In the *PtRab* family, four genes (*PtRabA1b*, *PtRabD1b*, *PtRabF2a* and *PtRabG3 g*) were not full-length (Additional file 2: Figure S1). *PtRabH1a* had a complete open reading frame, while it lost ~ 50 aa including the conserved motif-1 in the N-terminus, and the distance between motif-3 and motif-4 was much closer than the other PtRabs. Furthermore, a pseudogene *PtRabA1g.ψ* were identified which coding protein only retained 92 aa in N-terminus including motif-1.

### Chromosomal distribution and duplication among *PtRab* genes

**Fig. 2:**
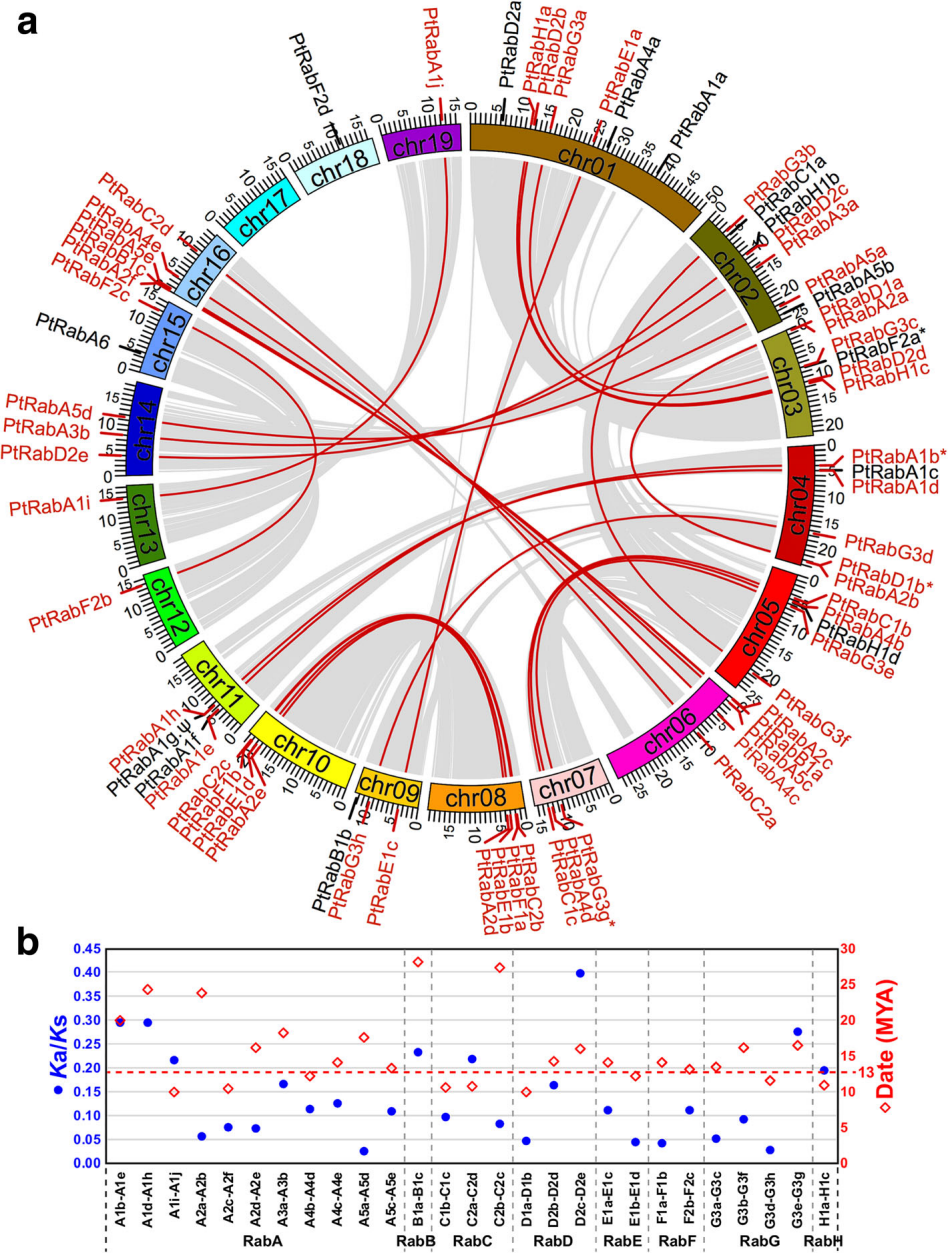
Genome-wide duplication of *PtRabs*. **a** Chromosome locations and duplication of *PtRabs*. Duplicated *PtRabs* were labelled as red and linked with red lines. Grey links between chromosomes indicate duplicated blocks generated by whole-genome duplication events. **b** *K*a/*K*s (left *y*-axis, blue dots) and duplication date (right *y*-axis, red dots) of pairwise *PtRabs* paralogous pairs

To explore the expansion mechanism of the *PtRab* family, we then analyzed its duplication patterns. All the 67 *PtRabs* unevenly distributed on 18 of 19 *Populus* chromosomes (Chr). Chr1 and Chr2 contain the most *Rab* genes (seven on each), while Chr17 does not contain any *Rab* gene (Fig. 2a). the *Populus* genome experienced at least two whole genome duplication (WGD) events and a series of chromosomal reorganizations [20]. In *PtRab* gene family, a total of 27 WGD paralogous pairs were identified, while no tandem duplication event was involved (Fig. 2 and Table 1). The nonsynonymous versus synonymous substitution rate ratios (*K*a/*K*s) were calculated to test whether Darwinian positive selection was involved in the *PtRab* gene divergence after duplication [22]. All the *K*a*/K*s ratios were prominent lower than 0.5 suggesting a purifying selection plays the dominate role in duplication of *PtRab* paralogous pairs (Fig. 2 and Table 1). Based on the divergence rate of λ = 9.1 × 10^− 9^ for *Populus* [23], the *PtRab* paralogous pairs were estimated to have occurred between 9.96 to 28.14 million years ago (MYA; Table 1). Recent large-scale genome duplication event in *Populus* occurred in ~ 13 MYA [20], all the *PtRab* paralogous pairs in five subfamilies (*RabD*-*RabH*) were generated around this stage, while *PtRabB1a*/*B1c* and *PtRabC2b*/*C2c* were generated in 28.14 and 27.32 MYA, respectively (Table 1 and Fig. 2b).

**Table 1:** Divergence between *PtRab* paralogous pairs

Subfamily	Gene 1	Gene 2	Duplication	*K*a	*K*s	*K*a/*K*s	Date (MYA)
RabA	*PtRabA1b*	*PtRabA1e*	W	0.108	0.366	0.295	20.13
	*PtRabA1d*	*PtRabA1h*	W	0.132	0.444	0.297	24.37
	*PtRabA1i*	*PtRabA1j*	W	0.040	0.182	0.218	9.98
	*PtRabA2a*	*PtRabA2b*	W	0.025	0.435	0.058	23.88
	*PtRabA2c*	*PtRabA2f*	W	0.014	0.191	0.075	10.50
	*PtRabA2d*	*PtRabA2e*	W	0.022	0.296	0.073	16.25
	*PtRabA3a*	*PtRabA3b*	W	0.056	0.334	0.167	18.35
	*PtRabA4b*	*PtRabA4d*	W	0.026	0.223	0.115	12.26
	*PtRabA4c*	*PtRabA4e*	W	0.032	0.257	0.126	14.10
	*PtRabA5a*	*PtRabA5d*	W	0.008	0.322	0.025	17.71
	*PtRabA5c*	*PtRabA5e*	W	0.027	0.243	0.110	13.35
RabB	*PtRabB1a*	*PtRabB1c*	W	0.120	0.512	0.235	28.14
RabC	*PtRabC1b*	*PtRabC1c*	W	0.019	0.193	0.098	10.59
	*PtRabC2a*	*PtRabC2d*	W	0.043	0.195	0.220	10.73
	*PtRabC2b*	*PtRabC2c*	W	0.042	0.497	0.084	27.32
RabD	*PtRabD1a*	*PtRabD1b*	W	0.009	0.181	0.047	9.96
	*PtRabD2b*	*PtRabD2d*	W	0.043	0.260	0.165	14.30
	*PtRabD2c*	*PtRabD2e*	W	0.117	0.293	0.399	16.12
RabE	*PtRabE1a*	*PtRabE1c*	W	0.029	0.258	0.112	14.15
	*PtRabE1b*	*PtRabE1d*	W	0.010	0.223	0.045	12.27
RabF	*PtRabF1a*	*PtRabF1b*	W	0.011	0.257	0.043	14.10
	*PtRabF2b*	*PtRabF2c*	W	0.027	0.239	0.111	13.14
RabG	*PtRabG3a*	*PtRabG3c*	W	0.013	0.246	0.051	13.54
	*PtRabG3b*	*PtRabG3f*	W	0.028	0.296	0.094	16.28
	*PtRabG3d*	*PtRabG3h*	W	0.006	0.212	0.029	11.63
	*PtRabG3e*	*PtRabG3g*	W	0.084	0.302	0.277	16.60
RabH	*PtRabH1a*	*PtRabH1c*	W	0.039	0.200	0.195	10.99

Notes: W, whole genome-wide duplication; *K*a, non-synonymous substitution rate; *K*s, synonymous substitution rate; MYA, million years ago

### Variety of *cis*-acting elements in the promoter regions of *PtRabs*

As transcription factors (TFs) binding sites, *cis*-acting elements located in gene promoter region are implicated in control of gene expression [24]. The 1.5 kb sequences upstream of translation start sites (TSS) of the *PtRab* genes were analyzed and the number of hormone responses and/or stress-related *cis*-acting elements were identified in the promoter regions of *PtRabs* (Additional file 4: Figure S3). Among the 67 *PtRabs*, 46 (68.7%) and 37 (55.2%) contain SA-responsive element (TCA-element) and MeJA-responsive elements (TGACG-motif or CGTCA-motif), respectively; and 24 *PtRabs* include both of them. Moreover, GA-, ABA-, ethylene- and auxin-responsive elements were found in promoter regions of 36, 30, 28 and 25 *PtRabs*, respectively. In addition, abundant stress-responsive elements were existed in the promoters of *PtRabs*. Top three stress-responsive elements were defense-, heat- and anoxia-responsive elements, which were presented in promoters of 54, 53 and 52 *PtRabs*, respectively (Additional file 4: Figure S3 and Additional file 5: Figure S4). These results indicated that *PtRab* genes might be involved in multiple hormone and stress responses.

### Differential expression profiles of *PtRabs* across tissues and under various stresses

To explore the potential roles of *PtRabs* in various tissues or developmental stages, the publicly available microarray data was used to analyze their expression patterns. Across various tissues, most of the *PtRab* members were highly expressed in phloem and xylem, especially *PtRabC* subfamily. In contrast, most of *PtRabs* were relatively low expressed in reproductive organs (Additional file 6: Figure S5A and Additional file 7: Table S2). During the stem development, more than half of *PtRabs* were highly expressed in the 5th and 9th internodes, which experience transition from primary growth to secondary growth (Additional file 6: Figure S5B). These results suggested *PtRab* genes might be involved in biological processes related with stem development.

We then investigated the expression profiles of *PtRabs* under various abiotic stresses, including low nitrogen, wounding, drought, heat, cold, and salinity stresses. Under low nitrogen treatment, only two *PtRabs* (*A3b* and *G3f*) were slightly reduced in expanding leaves under 8-week-treatment. To response mechanical wounding, 11 *PtRabs* (*A3a*, *A3b*, *A4b*, *A4d*, *A5a*, *A5b*, *A5d*, *C2a*, *D1a*, *G3e* and *G3f*) were up-regulated and 7 *PtRabs* (*A4a*, *A6*, *B1a*, *C1a*, *F1b*, *F2c* and *H1d*) were down-regulated in expanding leaved under 1-week-treatment. The expression of *PtRabs* were not sensitive to drought stress in both Soligo and Carpacio genotypes. Total of 14 *PtRabs* were affected under heat stress, which including 7 *PtRabs* (*A2c*, *A4a*, *A6*, *B1b*, *C1a*, *G3f* and *H1d*) were up-regulated and 7 *PtRabs* (*A3a*, *A5e*, *C2a*, *D1a*, *E1b*, *E1d* and *G3e*) were down-regulated. Under cold stress, two *PtRabs* (*A6* and *H1d*) and three *PtRabs* (*A3a*, *C2a* and *G3e*) were up- and down-regulated significantly (Additional file 6: Figure S5).

**Fig. 3:**
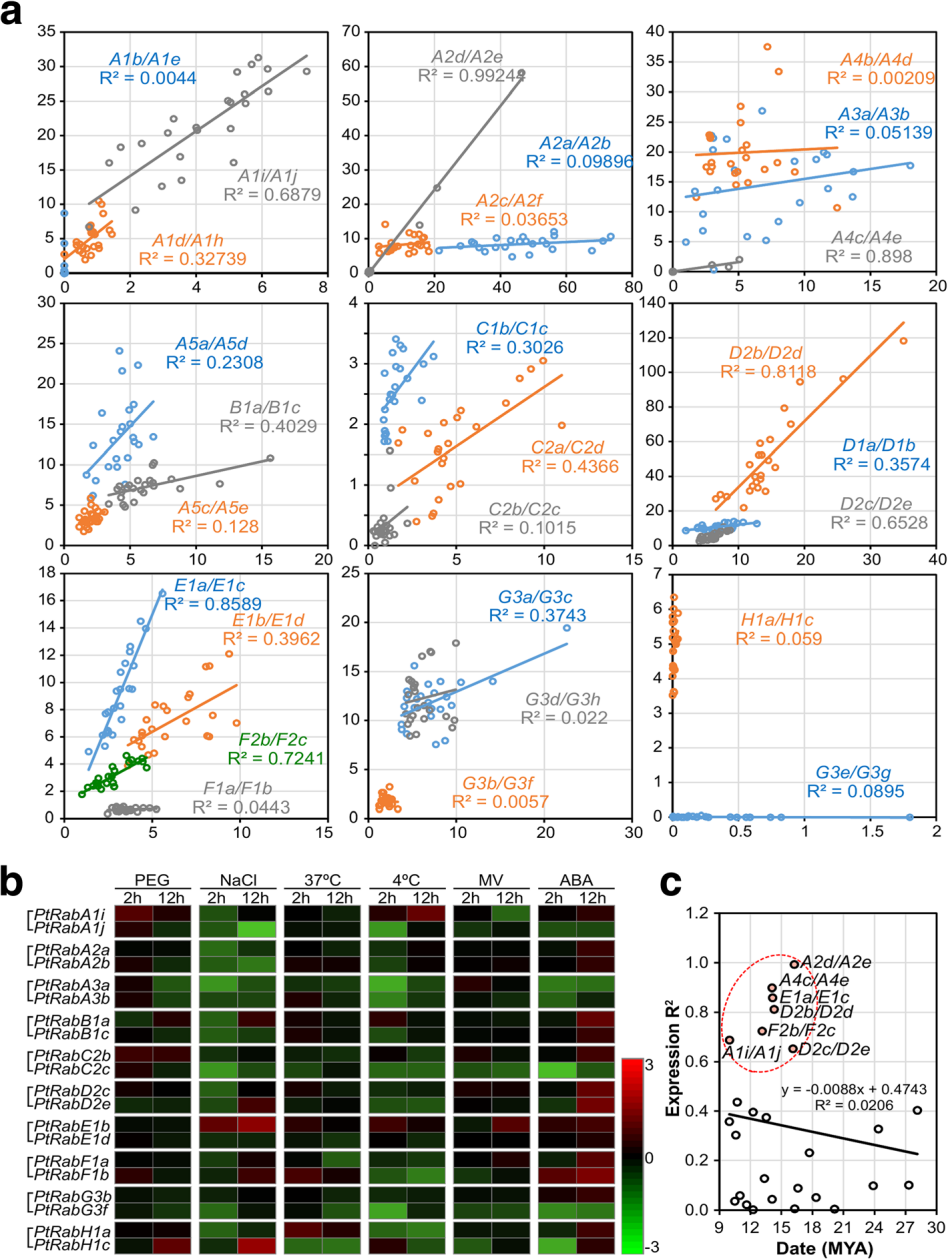
Expression analysis of 27 *PtRab* paralogous pairs. **a** Correlation of expression of 27 *PtRab* paralogous pairs across various tissues. *x*- and *y*-axis indicate FPKM values across 24 samples of two genes in each paralogous pair. For example, *x*-axis represents *PtRabA1b* and *y*-axis represents *PtRabA1e* in pair *PtRabA1b/A1e*. Expression values were shown in Table S3. **b** Expression validation of ten *PtRab* paralogous pairs response to various abiotic stresses using qRT-PCR. Red and green colors indicate up- and down-regulation of log2 transformed fold changes compared to control plants. **c** Correlation between duplication date and expression *R*^2^ of 27 *PtRab* paralogous pairs. Red dot circle includes seven pairs with highly similar patterns with *R*^2^ > 0.6

To reveal the divergence between *PtRab* paralogous pairs, the expression of 27 *PtRab* paralogous pairs were compared across 24 tissues. Only seven *PtRab* pairs (*A1i/A1j*, *A2d/A2e*, *A4c/A4e*, *D2b/D2d*, *D2c/D2e*, *E1a/E1c* and *F2b/F2c*) showed highly similar patterns with *R*^2^ > 0.6 across various tissues (Fig. 3a and Additional file 8: Table S3). This indicates most of the *PtRab* paralogous pairs were divergent during the evolution. Noticeably, four *PtRab* paralogous pairs (*A1b/A1e*, *F1a/F1b*, *H1a/H1c* and *G3e/G3 g*) showed significant differences (*R*^2^ < 0.1) in expression abundance between two genes in each pair - one has high abundance while another one was extremely low expressed in detected tissues (Fig. 3a). Expression *R*^2^ between genes in paralogous pairs was negatively correlated with their duplication date. Noticeably, the seven pairs with high expression *R*^2^ were duplicated during 9.98~ 16.25 MYA (red dot circle in Fig. 3c).

We then selected 10 *PtRab* paralogous pairs from eight subfamilies to validate their responsiveness under various abiotic stresses using qRT-PCR. Similar with the result from microarray data (Additional file 6: Figure S5), most genes in the 10 *PtRab* paralogous pairs were not sensitive to drought stress (Fig. 3b). Under salt, cold or oxidative stresses, most *PtRab* genes were down-regulated at 2 and 12 h after stress. While *PtRabE1b* and *PtRabH1c* were up-regulated under salt stress, especially *PtRabE1b* was prompt induced at both 2 and 12 h. This implying *PtRabE1b* might play a role in salt stress tolerance. Overall, the expression patterns of 10 selected *PtRab* paralogous pairs were divergent between the pairs in various abiotic stresses.

### Co-expression network of *PtRabs*

**Fig. 4:**
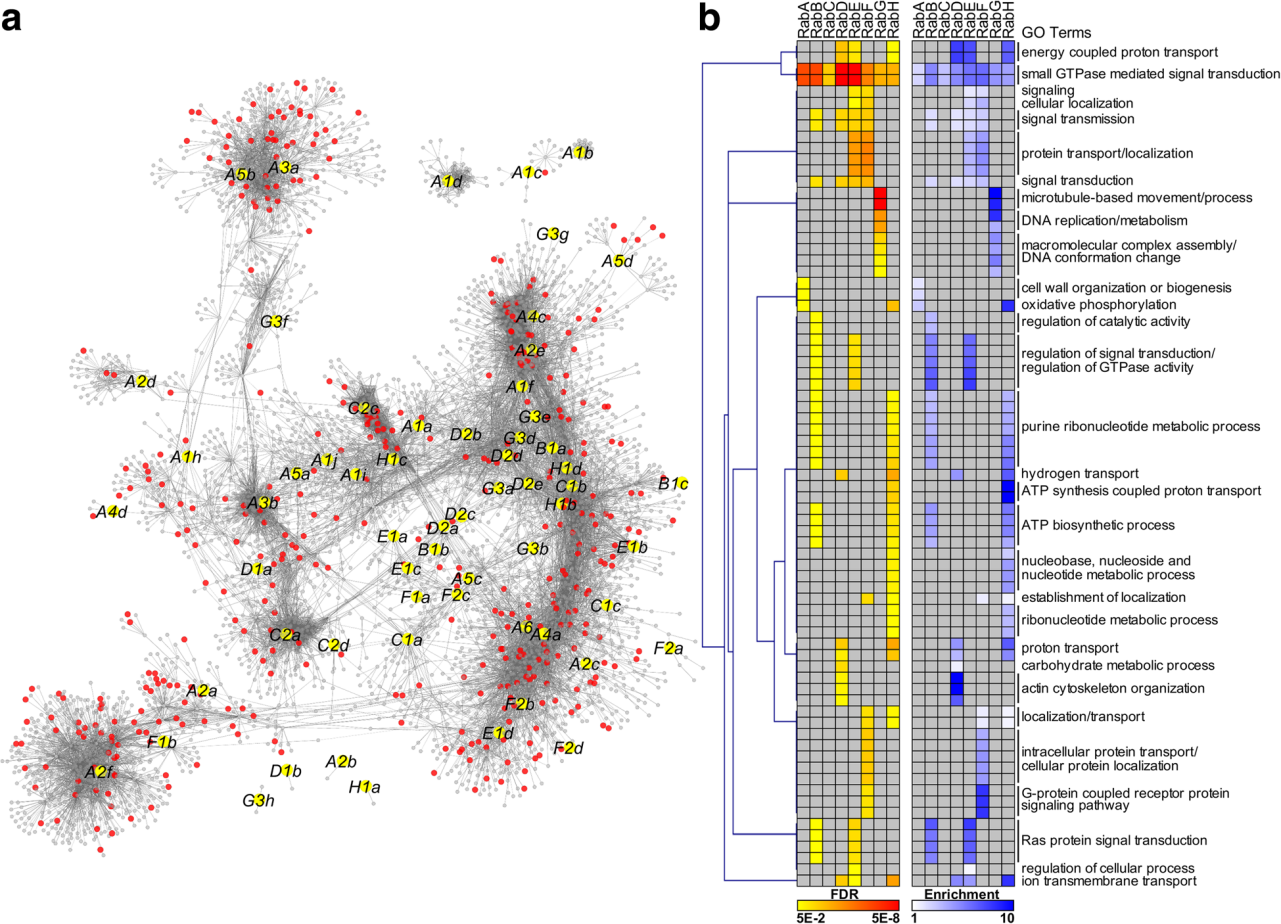
Co-expression network of *PtRabs*. **a** Co-expression network of *PtRabs*. Yellow and red nodes indicate *PtRabs* and transcription factors (TFs) in the co-expression network, respectively. **b** Hierarchical clustering of enriched GO terms of co-expression sub-networks of eight *PtRab* subfamilies (*RabA-H*). Colors represent FDR (yellow to red) or enrichment (white to blue); grey indicates no significant enrichment. Details of genes in *PtRabs* co-expression network and GO enrichment were shown in Table S4 and S5

To further reveal the functional differences of *PtRabs* in different subfamilies, we constructed a co-expression network of *PtRabs*. Total 61 of 67 *PtRabs* and 411 TFs were identified in the *PtRabs* co-expression network (Fig. 4a and Table S4). Among the 6151 genes in *PtRabs* co-expression network, *RabA* sub-network represents as the largest sub-network including a total of 4387 genes (71.32%) and 24 *RabA* members; whereas *RabF* and *RabG* sub-networks only including 6.15 and 7.79% in the whole network, respectively (Additional file 9: Figure S6). After screen the TF genes number (Additional file 9: Figure S6) and their enrichment (Additional file 10: Figure S7) in each sub-network, we found four *HSFs* (*HSFA4a*, *HSFA5a*, *HSFA5b* and *HSFB3b*) were enriched 7.697-fold in *RabE* sub-network.

Then the genes in sub-networks of different *PtRab* subfamilies were used for GO enrichment analysis. Noticeably, all the eight (*PtRabA-H*) sub-networks were enriched in “intracellular signaling cascade” and “small GTPase mediated signal transduction” (Fig. 4b, Additional file 11: Table S4, and Additional file 12: Table S5). *PtRabE* and *PtRabF* sub-networks were enriched in “signal process”, while *PtRabB* and *PtRabE* sub-networks were enriched in “regulation of GTPase activity”. For sub-network specific enriched GO terms, *PtRabA* sub-network was enriched in “cell wall organization”, *PtRabD* sub-network was enriched in “actin cytoskeleton organization”, *PtRabF* sub-network was enriched in “intracellular transport”, while *PtRabG* sub-network was enriched in “microtubule-based process” (Fig. 4b). In addition, the genes primarily co-expressed with *PtRabE1b* were functional classified based on their annotation. Total of 24 transport-, 11 stress-, 10 signal-, 5 protein-, 7 metabolic-, 6 development-, 4 cytoskeleton- and 2 autophagy-related genes were primarily co-expressed with *PtRabE1b* (Additional file 13: Figure S8).

### Subcellular localization of PtRab proteins in different subfamilies

**Fig. 5:**
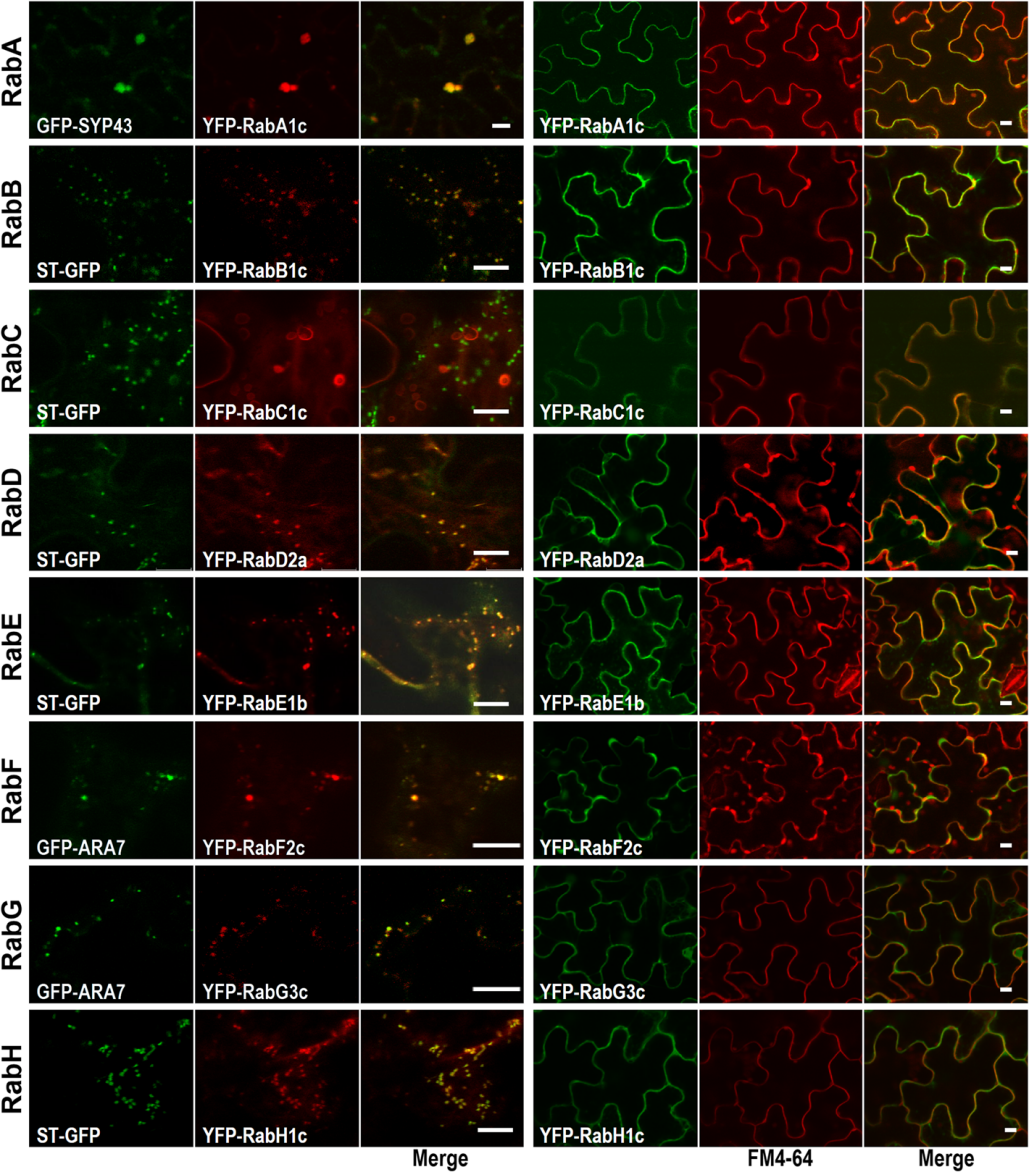
Subcellular localization of PtRab members in eight subfamilies. Confocal images of tobacco leaf epidermal cells co-expressing YFP-PtRabs (A1c, B1c, C1c, D2a, E1b, F2c, G3c and H1c) and different organelle markers (left panel) or FM4–64 (5 min after staining, right panel). Various organelle markers including GFP-SYP43 (for *trans*-Golgi network, TGN), ST-GFP (for Golgi) and GFP-ARA7 (for endosome). Bar, 10 μm

Plant Rab proteins coordinate different steps of endomembrane trafficking and showed specific subcellular localizations [1, 11]. Subsequently, eight PtRabs representing different subfamilies were selected for subcellular localization analysis in *Nicotiana benthamiana* leaf epidermal cells. The eight fusion proteins (*35S*::YFP-PtRabs) were detected on the PM as indicated by co-localized with the PM marker FM4–64 (Fig. 5 right panel). In addition, the well-known organelle markers including GFP-SYP43 (TGN), ST-GFP (Golgi), ARA7 (endosome) and GFP-HDEL (ER) were performed to confirm their precise localization in organelle. PtRabA1c was localized in the TGN, four PtRabs (B1c, D2a, E1b and H1c) were localized in the Golgi apparatus, while PtRabF2c and PtRabG3c were localized in the endosome (Fig. 5 left panel). However, PtRabC1c was localized in small vesicles but not co-localized with any organelle marker used in this study, its precise localization needs to be further determined.

### Natural variation of *PtRabE1b*

Based on the expression analysis, *PtRabE1b* was induced at 2 h after exposure to salt stress, and the induction was enhanced when the exposure was extended to 12 h (Fig. 3b). Furthermore, 11 stress-related genes were primarily co-expressed with *PtRabE1b* (Additional file 13: Figure S8). We then used *PtRabE1b* for further functional analysis. The natural variation of single nucleotide polymorphism (SNP) in a gene is known to introduce functional divergence in the gene. To detect if the conserved domains of PtRabE1b were affected in natural condition, the whole genome re-sequencing data of 549 *P. trichocarpa* natural individuals in North America were analyzed. As shown in Additional file 14: Figure S9, a total of 227 SNPs were identified in the *PtRabE1b* gene body (including UTR, exon and intron), and four SNPs affect non-synonymous coding (I45N, E100V, E168V and S201P). Noticeably, the four non-synonymous SNPs were not located in the conserved motifs of PtRabE1b (Additional file 14: Figure S9C).

### Overexpression of the constitutive activation mutant PtRabE1b(Q74L) confers salt tolerance in poplar

**Fig. 6:**
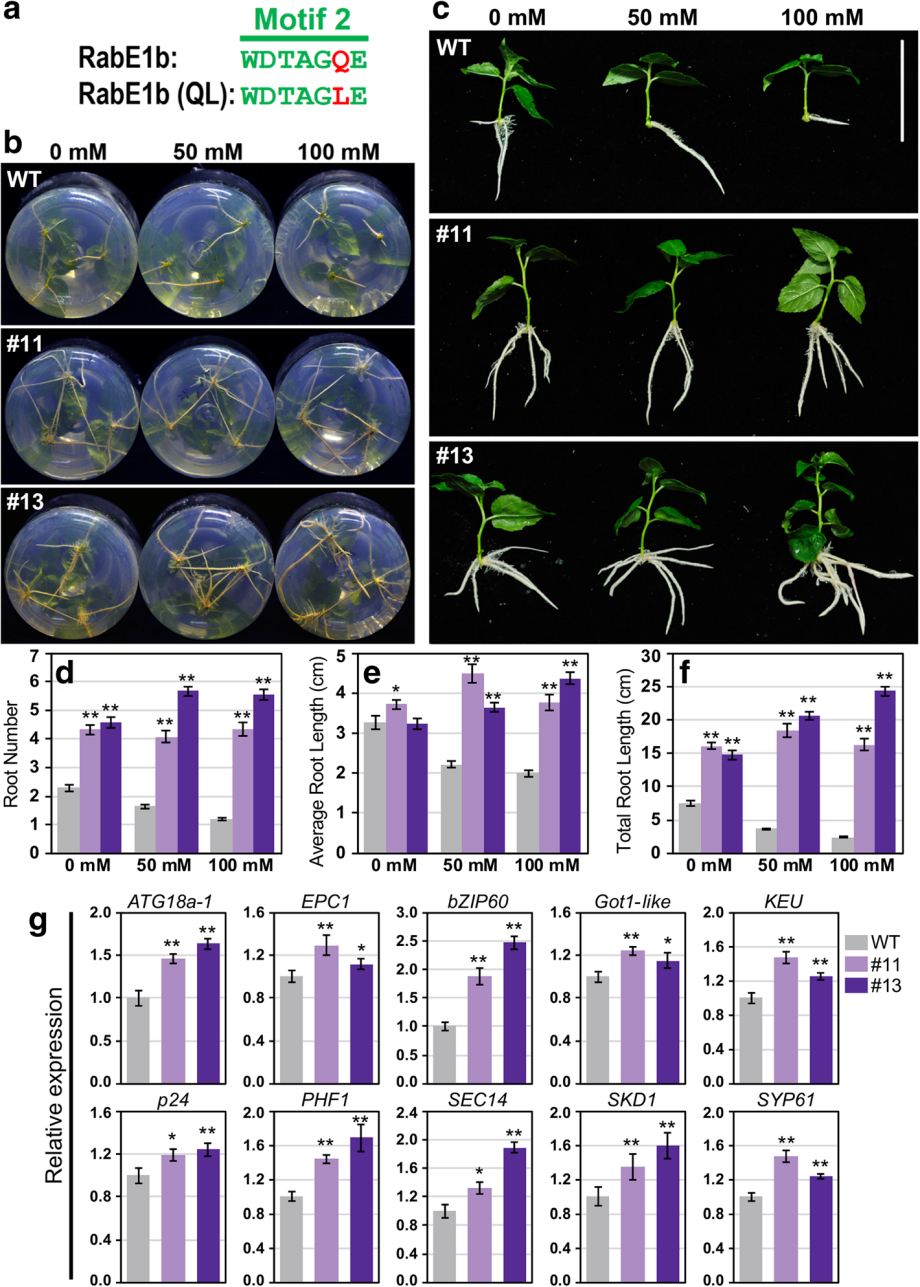
Overexpression of constitutive activation mutant of *PtRabE1b* confers salt tolerance in poplar. **a** Construction of a constitutively activation mutant of *PtRabE1b* at Q74L. **b, c** Growth status of wild type and two *PtRabE1b*(Q74L) overexpression lines (#11 and #13) under salt stress (0 mM, 50 mM and 100 mM NaCl). **d-f** Root number, average root length and total root length (*n* = 12). **g** Expression of selected co-expressed genes of *PtRabE1b* in wild type and *PtRabE1b*(Q74L) overexpression lines #11 and #13 under salt stress. Bars represent the mean ± SE of three independent experiments. * and ** represent significant differences at *P* < 0.05 and *P* < 0.01 compared with WT plants, respectively

To further investigate the function of *PtRabE1b*, a constitutively activate mutant PtRabE1b(Q74L) was constructed by point mutation of Q74 to L in motif 2 (Fig. 6a and Additional file 14: Figure S9C). A plant binary construct containing PtRabE1b(Q74L) driven by the CaMV *35S* promoter was generated and overexpressed in hybrid poplar (*P. alba* × *P. glandulosa*) clone 84 K. A total of 25 independent lines with hygromycin resistance were obtained and the positive transformants were confirmed using qRT-PCR. Two independent transgenic lines (#11 and #13) with the high transcript levels were selected for further analysis. Under normal condition, more adventitious roots (ARs) were differentiated in the two transgenic lines than wild type (WT), but the average root length was not significant differences between the transgenic lines and WT plants. When the seedlings were exposed to salt stresses (50 and 100 mM NaCl), both the root differentiation (root number) and root growth (root length) were inhibited in WT poplar. By contrast, the two transgenic lines maintained great root growth status even under salt stresses (Fig. 6). This indicates that overexpression the constitutive activate RabE1b(Q74L) enhanced salt tolerance in poplar. We then selected 10 different genes from the *PtRabE1b* co-expression network to validate their expression in PtRabE1b(Q74L) overexpression poplar. Seven trafficking-related genes (*Got1-like*, *KEU*, *p24*, *PHF1*, *SEC14*, *SKD1* and *SYP61*), a stress-related TF (*bZIP60*), an autophagy-related gene (*ATG18a-1*) and a development-related genes (*EPC1*) were up-regulated in the two PtRabE1b(Q74L) overexpression lines (Fig. 6g).

## Discussion

Rab GTPase as the important proteins in vesicular transport, are involved in the entire process of vesicle transport including budding from donor organelle, docking, tethering and fusing with the target membrane [3, 4]. Rab family was highly conserved in yeasts, mammalian and higher plants [6, 25], so it provide the consistent foundation for vesicle transport in organism. Here, a total of 67 non-redundant *Rab* genes were identified in *Populus*, it’s 1.2-fold than that in *Arabidopsis* (56 *AtRabs*) and relative lower than the ratio of 1.4~ 1.6 putative poplar homologs for *Arabidopsis* gene [20]. It seems that multicellutar organism has larger Rab GTPase family members compared to the 7–11 Rabs are found in the yeasts. It may be that multicellular organism is faced with complicated circumstance and adapt to complex metabolism.

The RabA subfamily is the largest subfamily in poplar and *Arabidopsis* (Fig. 1). However, yeast and animals only possess a few members corresponding to the RabA subgroup [25]. The Rab2 subgroup is closely related to human Rab11 and Rab25, which are generally thought to be associated with the polarize recycling of proteins at the PM. The studies on pea and tomato indicate RabAs play roles in the targeted secretion of cell-wall components to restricted areas of the cell surface [26]. In poplar, four more RabA members were identified than *Arabidopsis*, but its proportion among entire Rab family (41.8%; 28 *PtRabAs* of 67 *PtRabs*) is relative lower than that in *Arabidopsis* (42.9%; 24 of 56). Noticeably, the member of RabA6 was lack in poplar than *Arabidopsis*, which is similar with rice [27]; this implies RabA6 might not play conserved function across different species. During the expansion of the *Rab* family in the *Populus* genome, no tandem duplication event was observed. But there are two RabA clusters on Chr4 (PtRabA1b, PtRabA1c and PtRabA1d) and Chr11 (PtRabA1e, PtRabA1f and PtRabA1h), these two clusters might be undergone tandem duplication before the latest WGD. In contrast, the RabC subfamily was expanded to seven in poplar, while there are only three in *Arabidopsis* with unclear functions. In mammalian cells, its orthologue Rab18 is majorly involved in lipid droplet formation in the ER and possibly for cell metabolism in stressful conditions [28]. The expansion of RabC in poplar might be associated with the specific stress response or development, but it’s function still need further study. Based on the correlation analysis, only seven of 27 *PtRabs* paralogous pairs kept high correlation with *R*^2^ > 0.6, and about half (13 of 27) pairs showed significant divergence with *R*^2^ < 0.3 (Fig. 3). In addition, the expression *R*^2^ of *PtRab* paralogous pairs were negatively correlated with their duplication data. The seven *PtRab* paralogous pairs which still kept high expression similarity were mainly generated around the recent large-scale genome duplication event in *Populus* (~ 13 MYA; Fig. 3c) [20].

In the investigation of conserved Rab motifs, we found almost all the PtRab members had four conserved motifs, except several PtRabs which were not well annotated in the latest released *P. trichocarpa* genome. The four conserved motifs play crucial roles for their nucleotide binding and hydrolysis functions [29]. Three residues (S in motif 1, Q in motif 2 and N in motif 3 labelled in Additional file 2: Figure S1) could be used to generate the dominant negative or constitutively active mutants for gene functional investigation. In this study, we constructed an active mutation of PtRabE1b through point mutation of Q74 to L in motif 2 and overexpressed PtRabE1b(Q74L) in poplar. The phenotypes of PtRabE1b(Q74L)-overexpression poplars under normal condition and salt stress proved mutation at Q74 of PtRabE1b can introduce constitutive activity and this mutation can be used for gene functional studies.

The Rab proteins exhibit restricted and specific subcellular localizations. Mammalian Rab11 resides on recycling endosomes and regulates traffic to specific PM domains and to the TGN in diverse cell types [30]. The function of Rab11 orthologous may be conserved either in mammalian or plant. As the orthologous of Rab11 in plants, RabA subfamily members have the same localization. RabA1a, RabA1b and RabA1c co-localized with TGN marker in the division zone of roots [8]. Moreover, members in RabA subfamily had evolved divergent function in distinct steps of subcellular trafficking. In poplar, *PtRabA1c* was precisely located to TGN and PM, which implied the functional conservation of RabA subfamily. Either transient expression in tobacco epidermal cell or stable expression in *Arabidopsis* indicate AtRabE1d localized in Golgi apparatus and PM [11, 12], which were consistent with our findings of PtRabE1b. For members in other subfamilies, most of them showed similar localization with the orthologous reported in other plant species [13]. Whereas, the localization evidence of RabC is still scarce. A study using mammalian cells reveals Rab18, an orthologous of RabC, was localized to endosomes through antibody staining. In addition, it was also existed in ER and Golgi apparatus [28]. In our study, PtRabC1c was localized in small vesicles, but it was not overlapped with any marker except PM. Further studies should be conducted to survey the various localization of Rab protein.

Rab GTPases have various roles in development. *Arabidopsis RabE* down-regulation resulted in drastically altered leaf morphology and reduced plant size [12]. Tobacco *NbRabE1* silenced resulted in growth arrest, premature senescence, and abnormal leaf development. The dominant negative mutant of *NbRabE1* in *Arabidopsis* resulted in retardation of shoot and root growth accompanied by defective root hair formation [31]. Except members in *RabE* subfamily, *Rab* genes in other subfamilies were also play important roles. Overexpression a constitutively active form of *Arabidopsis RabG3b* promotes xylem development through the activation of autophagy in the transgenic poplar [17]. Among the processes unique to tree biology, one of the most obvious is the yearly development of secondary xylem from the vascular cambium different from herbaceous plants. From our results, almost all the *Rab* genes were highly expressed in xylem and phloem. Moreover, the expression level of *Rab* genes were elevated following the maturity of internode (Additional file 6: Figure S5). It can explain massive materials’ transport within cell during this developmental process. Generally, wood is a product of plant cell wall polymers and wood formation is the consequences of plant cell wall deposition. Various plant cell wall synthesis-related enzymes and metabolites have been shown to be mediated by membrane trafficking. For instance, cellulose synthase, hemicellulose, pectin and other polysaccharides transport from Golgi apparatus to cell membrane or secrete to extracellular space [32–34].

As sessile organisms, plant have acquired different strategies to respond to various biotic and abiotic stresses in their ambient environment [35]. During the stress response, *cis*-acting elements play pivotal roles in regulating genes expression by controlling TF binding site and promoter efficiency [24]. Our results indicated that the *cis*-acting elements of *PtRab* genes were involved in multiple hormone and stress responses. A majority of *PtRab* promoters contain SA- and MeJA-responsive elements, which implied *PtRab* genes might be involved in pathogen resistance. In tomato, two RabE members Api2 and Api3 can interact with the *Pst DC3000* TTSS effector AvrPto [36]. Overexpression of the constitutively active RabE1d(Q74L) conferring *Arabidopsis* resistance to *P. syringae* infection [12]. In addition, *Rab* genes are involved in abiotic stress tolerance in plants. For instance, both the *Arabidopsis RabA1* quadruple mutant (completely knocked out for expression of *RabA1a*, *A1b*, *A1c* and *A1d*) and expressing the dominant-negative mutant of RabA1b(S27b) exhibited hypersensitivity to salinity stress at 15 mM NaCl concentration [8]. Loss-of-function of *ARA6* (also known as *RabF1*) conferred *Arabidopsis* salt hypersensitivity; but overexpression of ARA6(Q93L)-GFP conferred tolerance to high-salt stress, which formed discrete speckles on the PM after salt stress with no other PM proteins were affected [37]. Moreover, overexpression of *Pennisetum glaucum PgRab7* or *Prosopis juliflora PjRab7*, which are homologs of *Arabidopsis RabG3e*, confer salt tolerance to transgenic tobacco [19, 38]. In plants, Rabs are expanded greatly but the function of many of them is unknown. This may reflect the fact that Rabs are expanded to deal with prevailing environmental conditions, which was supported by our *cis*-acting elements, co-expression network and expression profiles analyses. In our study, overexpression of constitutively active RabE1b(Q74L) confer salt tolerance in transgenic poplar (Fig. 6). From the co-expression network, four *HSF* including *HSFA4a* were highly enriched in *RabE* sub-network (Fig. 4 and Additional file 10: Figure S7). In *Arabidopsis*, *HSFA4a* confers salt tolerance and regulated by oxidative stress and MPK3/MPK6 [39]. It’s homologue in *Chrysanthemum*, *CmHSFA4*, was also been proved positively regulate salt tolerance through maintain Na^+^/K^+^ ion balance with *SOS1* and *HKT2* and regulate ROS scavenger activities [40]. Among the genes directly co-expressed with *PtRabE1b* (Additional file 13: Figure S8), *SOS2*, *MPK19* and Ca^2+^ signaling related genes (*CAM7*, *CKL6* and *calcium exchanger*) were consistent with these studies on plant salt tolerance. The *SOS2* encodes a serine/threonine protein kinase with an amino-terminal catalytic domain and a carboxy-terminal regulatory domain. SOS2 can physically interact with and be activated by SOS3, a myristoylated calcium-binding protein, to form SOS3-SOS2 kinase complex for transcriptional regulation of *SOS1*. And this pathway was mediated by calcium signal [41]. Mitogen-activated protein kinase (MAPK) cascades are universal signal transduction modules in eukaryotes. Several members in MPK family have been reported were involved in salt stress response, e.g. MPK3, MPK4 and MPK6 are activated by NaCl stress, expression of active MKK9 through activation of endogenous MPK3 and MPK6 enhances salt sensitivity in *Arabidopsis* [42]. Moreover, MPK6 can phosphorylates the C–terminal fragment of SOS1 to exclude Na^+^ from cells [43].

## Conclusions

In conclusion, we comprehensively analyzed the evolutionary relationship of the *Rab* gene family in *Populus* and functionally characterized *PtRabE1b*, which confers salt tolerance in transgenic poplar. Our findings provide a deeper insight of the structure-localization-function relationships of vesicular transport genes and an abundant resource for genetic engineering to improve stress tolerance in trees.

## Methods

### Sequence retrieval and phylogenetic reconstruction

Published *Arabidopsis Rab* sequences [1] were retrieved and were used as queries in BLAST searches against the *P. trichocarpa* genome database (http://phytozome.jgi.doe.gov/pz/portal.html#!info?alias=Org_Ptrichocarpa) to identify potential *PtRab* genes. All homologous protein sequences of the predicted Rab family members were accepted if they were satisfied with expectation (*E*) value < 10^− 10^. Multiple alignment of Rab proteins from *P. trichocarpa* and *A. thaliana* were performed using the Clustal X2.1 [44]. The maximum likelihood (ML) phylogenetic trees were constructed using MEGA 7.0.26 [45] under the Jones-Taylor-Thornton (JTT) amino acid substitution model with 1000 bootstraps.

### Bioinformatics analyses of *Populus Rab* genes

The exon and intron structures were illustrated using Gene Structure Display Server (GSDS) [46]. The chromosomal locations and duplication of the *Rab* genes were drawn using Circos software [47]. To analyze the putative *cis*-acting regulatory elements, 1500 bp upstream of translation start site (TSS) were searched using PlantCARE database [24]. The PAL2NAL program (http://www.bork.embl.de/pal2nal/) was used to estimate synonymous (*K*s) and nonsynonymous (*K*a) substitution rates. The divergence time (T) was calculated according to T = *K*s/(2 × 9.1 × 10^− 9^) million years ago (MYA) for *Populus* [23]. For co-expression network construction, the expression data was obtained in the *Populus* Gene Atlas Study from Phytozome (https://phytozome.jgi.doe.gov/pz/portal.html#). Pearson correlations were calculated in parallel between all pairs of gene expression vectors. A threshold greater than or equal to 0.85 was applied to resulting correlations and the remaining correlations were visualized by Cytoscape [48]. GO enrichment of each sub-network was performed using agriGO [49].

### Publicly available microarray data analyses

The microarray data for various tissues and developmental stages were obtained from the NCBI Gene Expression Omnibus (GEO) database with the accession numbers GSE21481, GSE21485 and GSE13043. For abiotic stresses, Affymetrix microarray data with the series accession numbers GSE14893 and GSE148515 (nitrogen limitation), GSE16783 and GSE16785 (wounding), GSE17225 (drought), GSE41557 (heat, 42 °C 6 h) and GSE43872 (cold, 4 °C 10 h) were analyzed. Probe sets corresponding to *PtRabs* genes were identified using the online Probe Match tool POParray (http://aspendb.uga.edu) and were listed in Additional file 3: Table S2. The data was normalized using the Gene Chip Robust Multiarray Analysis (GCRMA) algorithm followed by log transformation and average calculations. For correlation analysis of 27 *PtRab* paralogous pairs, the expression data of *PtRabs* across 24 *P. trichocarpa* tissues were obtained in the *Populus* Gene Atlas Study from Phytozome (https://phytozome.jgi.doe.gov/pz/portal.html#). Pearson correlations were calculated between paralogous genes in each pair.

### Plasmids and constructs

The full-length CDS of *PtRabA1c*, *RabB1c*, *RabC1c*, *RabD2a*, *RabF2c*, *RabG3c* and *RabH1c* were amplified from the cDNA of *P. trichocarpa* and was introduced into the pDNOR222.1 (Life technologies, Carlsbad, California, U.S.) to produce pENTR vectors. Subsequently, the genes in the pENTR vectors were validated by sequencing and then subcloned into pEarleyGate104 (ABRC stock DB3–686) to produce *35S*::YFP-*PtRab*s constructs using the Gateway cloning system (Invitrogen). To generate Q74L substitution of PtRabE1b, site-directed mutagenesis was conducted by overlapping PCR. Four primers containing the desired changes (underlined) were used: PtRabE1b-QL-1, 5’-ATGGCTGCACCGCCAG-3′; PtRabE1b-QL-2, 5’-AGTTCGGAAACGTTCC**A**GGCCTGCTGTATCCA-3′; PtRabE1b-QL-3, 5’-TGGGATACAGCAGGCC**T**GGAACGTTTCCGAACT-3′; PtRabE1b-QL-4, 5’-TTATGAACCACAGCAAGCTGACT-3′. Through a change at 221 bp of *PtRabE1b* CDS (A to T), PtRabE1b(Q74L) was obtained. The coding sequences of PtRabE1b(Q74L) was introduced into pDNOR222.1 and subcloned into pMDC32 using the Gateway cloning system (Invitrogen). The expression vectors were transferred into *Agrobacterium tumefaciens* GV3101 by electroporation.

### Plant material, growth conditions and transformation

One-year-old *P. trichocarpa* grown in a growth chamber under long-day conditions (16/8 h light/dark) at 23–25 °C. Plant materials for qRT-PCR in different tissues (YL, young leaf; ML, mature leaf; PS, primary stem; SS, secondary stem; R, root.) were collected from hybrid poplar (*P. alba* × *P. glandulosa*) clone 84 K. Samples were frozen immediately in liquid nitrogen, and stored at − 80 °C for further analysis. Three biological replicates were performed.

For abiotic stress and hormone treatments, *P. trichocarpa* seedlings were water-cultured in Hoagland’s nutrient solution for 15 days after subculture on 1/2 MS solid medium. Then the seedlings were treated with 10% polyethylene glycol (PEG, for drought stress), 150 mM NaCl (for salt stress), 37 °C (for heat stress), 4 °C (for cold stress), 10 μM methyl viologen (MV, for oxidative stress) or 100 μM abscisic acid (ABA), respectively. The control plants were grown in Hoagland’s nutrient solution under normal condition. During the treatments, three time-points (0, 2 and 12 h) were chosen for samples collection. The above samples were immediately frozen in liquid nitrogen after harvest and stored at − 80 °C for further analysis.

Poplar stable transformation of PtRabE1b(Q74L) was through leaf-disc method as described by Liu et al. [50]. A total of 25 independent transgenic lines carrying the *35S*::PtRabE1b(Q74L) construct with hygromycin resistance were obtained. Two lines (#11 and #13) with high transcript levels of PtRabE1b(Q74L) were used for further analysis.

### Subcellular localization of PtRabs

Co-expression of *35S*::YFP-*PtRab*s constructs and fluorescent markers in *Nicotiana benthamiana* leaf lower epidermal cells was performed using *Agrobacterium* transformation as described by Zhang et al. [51]. The fluorescence of infiltrated tobacco leaves was observed using a LSM 510 confocal laser scanning microscope. For fluorescence imaging of YFP/FM4–64 samples, 488/543-nm excitation and 505–530/585-nm filters were used. For GFP/YFP samples, 458/514-nm excitation and 475–525/520-555 nm filters were used.

### RNA isolation and real-time qRT-PCR

Total RNA was extracted using the RNeasy Plant Mini Kit (Qiagen) with on-column treatment with RNase-free DNase I (Qiagen) to remove genomic DNA. First-strand cDNA synthesis was carried out with approximately 1 μg RNA using the SuperScript III reverse transcription kit (Invitrogen) according to the manufacturer’s procedure. Primers with melting temperatures of 58–60 °C and amplicon lengths of 100–250 bp were designed using Primer3 software (http://frodo.wi.mit.edu/primer3/input.htm). All primer sequences are listed in Additional file 15: Table S6. Real-time qRT-PCR was performed in quadruplicate using the SYBR Premix Ex Taq™ II Kit (TaKaRa, Dalian, China) on a Roche LightCycler 480 (Roche Applied Science, Penzberg, Upper bavaria, Germany) according to the manufacturer’s instructions. All experiments were performed in three biological replicates and four technical replicates. The *PtActin* and *PtTubulin* genes were used as internal controls.

### Statistical analyses

All data are presented as the means ± standard error (SE) of at least three replicates. The Student’s *t*-test was used to test the significance of differences between the control plants and transgenic lines. Asterisks (* or **) indicate a significant difference between the control and transgenic plants at *P* < 0.05 or 0.01, respectively.

## Additional files

Additional file 1: **Table S1.** Rab gene families in *P. trichocarpa*. (DOCX 24 kb)

Additional file 2: **Figure S1.** Gene structures and motif composition of *PtRabs*. (A) The exon-intron structures of *PtRab* genes. The numbers (0, 1 and 2) indicate the splicing phases (phase 0, 1 and 2, respectively) of the Rab genes. (B) A schematic representation of conserved motifs in Rab proteins. Four conserved motifs (1–4) are represented by different colors. (TIF 3329 kb)

Additional file 3: **Figure S2.** Conserved motifs and their sequences in PtRab proteins. The four motifs are involved in nucleotide binding and hydrolysis. Mutation of the residues (shown in red asterisk) could be used to generate dominant-negative and constitutively active forms that exhibit altered nucleotide-binding or hydrolysis characteristics, which could be used to investigate Rab function. (TIF 1105 kb)

Additional file 4: **Figure S3.** The number of *Rab* genes containing various *cis*-acting elements. Auxin-responsive elements: TGA-element, TGA-box and AuxRR-core; gibberellin-responsive elements: TATC-box, P-box and GARE-motif; abscisic acid (ABA)-responsive elements: CE3 and ABRE; salicylic acid (SA)-responsive element: TCA-element; MeJA-responsive elements: TGACG-motif and CGTCA-motif; ethylene-responsive element: ERE; heat-responsive element: HSE; cold-responsive element: LTR and C-repeat/DRE; MYB binding site involved in drought-inducibility: MBS; defense-related elements: Box-W1 and TC-rich repeats; anoxic-related elements: GC-motif and ARE; wound-responsive element: WUN-motif. (TIF 415 kb)

Additional file 5: **Figure S4.** Various *cis*-acting elements in responsive to hormone and stresses in *Rab* promoters. (TIF 1113 kb)

Additional file 6: **Figure S5.** Expression profiles of *PtRab* genes across different tissues and under various abiotic stresses. Heatmap showing expression of *PtRab* genes across tissues (A), different stem development/growth stages (B) and under various abiotic stresses (C) in poplar. For abiotic stresses, Stress treatments including: low N, nitrogen limitation; wounding, sampled either one week or 90 h after wounding; EAR, early response to water deficit by 36 h; LMI, long-term (10 day) response to mild stress with soil relative extractable water (REW) at 20–35%; LMO, long-term (10 day) response to moderate stress with soil REW at 10–20%; heat, 42 °C for 6 h; and cold, 4 °C for 10 h. Genotypes including *P. fremontii* × *P. angustifolia* clones 1979, 3200 and RM5 (low N and wounding), *P. deltoids* clones Soligo and Carpaccio (drought), and *P. simonii* (heat and cold). Color scale represents log2 expression values, green represents low level and red indicates high level of transcript abundances. Blank represents a gene has no corresponding probe sets in the microarray data. The microarray data (GSE21481, GSE21485, GSE13043, GSE16786, GSE17230, GSE41557 and GSE43872) were obtained from NCBI Gene Expression Omnibus (GEO) database. (TIF 3420 kb)

Additional file 7: **Table S2.** Probe sets corresponding to *PtRab* genes. (DOCX 22 kb)

Additional file 8: **Table S3.** Expression data of 27 *PtRab* paralogous pairs used for correlation analysis. (XLSX 22 kb)

Additional file 9: **Figure S6.** Summary of Rab and TF subfamilies gene numbers in *PtRabs* co-expression sub- networks. (TIF 945 kb)

Additional file 10: **Figure S7.** TF enrichment of different subfamilies in *PtRabs* co-expression sub-networks. (TIF 1210 kb)

Additional file 11: **Table S4.** Functional annotation of genes in *PtRabs* co-expression network. (XLSX 484 kb)

Additional file 12: **Table S5.** Enriched GO terms of genes in *PtRabs* sub-networks. (XLSX 16 kb)

Additional file 13: **Figure S8.** Functional classification of genes primarily co-expressed with *PtRabE1b*. (TIF 1141 kb)

Additional file 14: **Figure S9.** Natural variation and protein structure of PtRabE1b. (A) Identification of SNPs in *PtRabE1b* from 549 *P. trichocarpa* individuals. (B) Effects of SNPs located in *PtRabE1b*. (C) Protein structure of PtRabE1b. Four non-synonymous SNPs affected proteins (I45N, E100V, E168V and S201P) were labelled as yellow. Q74L in motif-2 used for constitutive activation was labelled as green. (TIF 631 kb)

Additional file 15: **Table S6** qRT-PCR Primers used in this study. (DOCX 18 kb)

## Abbreviations

ABA: Abscisic acid
ER: Endoplasmic reticulum
HVD: Hypervariable C-terminal domain
*K*a: nonsynonymous substitution rate
*K*s: synonymous substitution rate
MV: Methyl viologen
MYA: Million years ago
PEG: Polyethylene glycol
SNARE: the soluble N-ethylmaleimide-sensitive factor attachment receptors
SNP: Single nucleotide polymorphism
TF: Transcription factor
TGN: Trans-Golgi network
TSS: Translation start site
UTR: Untranslated region
WGD: Whole genome duplication
YFP: Yellow fluorescent protein
