# Supplementary material for: Characterization of the Populus Rab family genes and the function of PtRabE1b in salt tolerance
Source: BMC Plant Biol. 2018 Jun 18;18:124. doi: 10.1186/s12870-018-1342-1 (PMC6006591; doi:10.1186/s12870-018-1342-1)
Supplement: Supplementary file 1 — Table S1. Rab gene families in P. trichocarpa. (DOCX 24 kb) [file 12870_2018_1342_MOESM1_ESM.docx]

### Table S1. Rab gene families in *P. trichocarpa*.

| **Subfamilies** | **Subgroups** | **Gene Name** | **Gene Locus** | **Location** | **CDS (bp) /**  **ORF (aa)** | **pI / MW (kDa)** |
| --- | --- | --- | --- | --- | --- | --- |
| **RabA** | **A1** | *PtRabA1a* | Potri.001G374000 | Chr01: 38848029-38850871 (-) | 648 / 215 | 24.2 / 5.48 |
|  |  | *PtRabA1b** | Potri.004G051400 | Chr04: 3980745-3981235 (-) | 417 / 138 | 15.5 / 6.34 |
|  |  | *PtRabA1c* | Potri.004G060500 | Chr04: 4898482-4902963 (+) | 657 / 218 | 24.2 / 5.51 |
|  |  | *PtRabA1d* | Potri.004G061000 | Chr04: 4976078-4980535 (+) | 657 / 218 | 24.2 / 5.51 |
|  |  | *PtRabA1e* | Potri.011G060900 | Chr11: 5447638-5449627 (-) | 654 / 217 | 24.3 / 5.72 |
|  |  | *PtRabA1f* | Potri.011G061300 | Chr11: 5460321-5462305 (-) | 654 / 217 | 24.3 / 5.72 |
|  |  | *PtRabA1g.ψ* | Potri.011G070200 | Chr11: 6597209-6597697 (+) | 276 / 91 | 10.4 / 4.95 |
|  |  | *PtRabA1h* | Potri.011G070300 | Chr11: 6607768-6612140 (+) | 657 / 218 | 24.2 / 5.53 |
|  |  | *PtRabA1i* | Potri.013G123600 | Chr13: 13740209-13742283 (-) | 678 / 225 | 25.3 / 5.99 |
|  |  | *PtRabA1j* | Potri.019G092500 | Chr19: 12304881-12306982 (+) | 651 / 216 | 24 / 5.59 |
|  | **A2** | *PtRabA2a* | Potri.003G004100 | Chr03: 369776-370995 (-) | 654 / 217 | 24 / 6.76 |
|  |  | *PtRabA2b* | Potri.004G226400 | Chr04: 23208875-23214250 (+) | 648 / 215 | 23.8 / 6.21 |
|  |  | *PtRabA2c* | Potri.006G000300 | Chr06: 30533-32605 (+) | 651 / 216 | 23.9 / 6.31 |
|  |  | *PtRabA2d* | Potri.008G061300 | Chr08: 3695827-3697846 (-) | 651 / 216 | 24 / 6.83 |
|  |  | *PtRabA2e* | Potri.010G197200 | Chr10: 19010653-19012438 (+) | 651 / 216 | 24 / 7.68 |
|  |  | *PtRabA2f* | Potri.016G000400 | Chr16: 26045-28526 (-) | 651 / 216 | 23.8 / 6.31 |
|  | **A3** | *PtRabA3a* | Potri.002G175700 | Chr02: 13501179-13502928 (-) | 705 / 234 | 25.9 / 4.8 |
|  |  | *PtRabA3b* | Potri.014G102200 | Chr14: 7991475-7992934 (+) | 717 / 238 | 26.4 / 4.9 |
|  | **A4** | *PtRabA4a* | Potri.001G270100 | Chr01: 27745149-27748072 (+) | 678 / 225 | 24.8 / 5.98 |
|  |  | *PtRabA4b* | Potri.005G073000 | Chr05: 5322602-5325464 (-) | 672 / 223 | 24.6 / 7.73 |
|  |  | *PtRabA4c* | Potri.006G057700 | Chr06: 4215027-4217174 (+) | 657 / 218 | 24.5 / 7.65 |
|  |  | *PtRabA4d* | Potri.007G096000 | Chr07: 12210373-12213103 (+) | 672 / 223 | 24.6 / 6.84 |
|  |  | *PtRabA4e* | Potri.016G050400 | Chr16: 3206587-3211979 (-) | 657 / 218 | 24.6 / 7.68 |
|  | **A5** | *PtRabA5a* | Potri.002G231800 | Chr02: 22420255-22425150 (+) | 654 / 217 | 24.2 / 5.14 |
|  |  | *PtRabA5b* | Potri.002G249500 | Chr02: 24015016-24017482 (-) | 657 / 218 | 24.3 / 4.85 |
|  |  | *PtRabA5c* | Potri.006G015400 | Chr06: 1056760-1059129 (+) | 678 / 225 | 24.7 / 5.8 |
|  |  | *PtRabA5d* | Potri.014G150300 | Chr14: 11535266-11540096 (-) | 654 / 217 | 24.3 / 5.14 |
|  |  | *PtRabA5e* | Potri.016G010300 | Chr16: 516797-519966 (-) | 675 / 224 | 24.7 / 5.22 |
|  | **A6** | *PtRabA6* | Potri.015G039700 | Chr15: 3630818-3633725 (+) | 669 / 222 | 25.1 / 4.98 |
| **RabB** | **B1** | *PtRabB1a* | Potri.006G001500 | Chr06: 136498-139080 (-) | 636 / 211 | 23.1 / 6.96 |
|  |  | *PtRabB1b* | Potri.009G159600 | Chr09: 12331652-12334022 (-) | 726 / 241 | 27.6 / 8.72 |
|  |  | *PtRabB1c* | Potri.016G002200 | Chr16: 107417-110070 (-) | 645 / 214 | 24.3 / 7.77 |
| **RabC** | **C1** | *PtRabC1a* | Potri.002G074400 | Chr02: 5140604-5143365 (+) | 636 / 211 | 23.8 / 6.01 |
|  |  | *PtRabC1b* | Potri.005G063500 | Chr05: 4567708-4571594 (+) | 630 / 209 | 23.4 / 5.69 |
|  |  | *PtRabC1c* | Potri.007G105500 | Chr07: 12979263-12983866 (-) | 630 / 209 | 23.4 / 6.39 |
|  | **C2** | *PtRabC2a* | Potri.006G121400 | Chr06: 9671558-9675215 (+) | 642 / 213 | 23.4 / 5.8 |
|  |  | *PtRabC2b* | Potri.008G032000 | Chr08: 1749697-1752703 (+) | 645 / 214 | 23.6 / 8.53 |
|  |  | *PtRabC2c* | Potri.010G229600 | Chr10: 21200754-21203155 (-) | 639 / 212 | 23.7 / 8.47 |
|  |  | *PtRabC2d* | Potri.016G097800 | Chr16: 9462102-9465647 (+) | 627 / 208 | 22.9 / 6.33 |
| **RabD** | **D1** | *PtRabD1a* | Potri.003G004000 | Chr03: 365938-369609 (+) | 606 / 201 | 22.5 / 5.01 |
|  |  | *PtRabD1b** | Potri.004G226600 | Chr04: 23215030-23218609 (-) | 756 / 251 | 28 / 5.23 |
|  | **D2** | *PtRabD2a* | Potri.001G080400 | Chr01: 6371996-6376473 (-) | 612 / 203 | 22.6 / 5.27 |
|  |  | *PtRabD2b* | Potri.001G152800 | Chr01: 12609556-12611546 (+) | 609 / 202 | 22.4 / 6.58 |
|  |  | *PtRabD2c* | Potri.002G138400 | Chr02: 10284548-10287825 (+) | 636 / 211 | 23.6 / 5.39 |
|  |  | *PtRabD2d* | Potri.003G081800 | Chr03: 10967025-10969631 (-) | 612 / 203 | 22.7 / 5.54 |
|  |  | *PtRabD2e* | Potri.014G049400 | Chr14: 3968268-3971682 (-) | 612 / 203 | 22.6 / 5.12 |
| **RabE** | **E1** | *PtRabE1a* | Potri.001G236100 | Chr01: 24776751-24779863 (-) | 651 / 216 | 23.8 / 8.38 |
|  |  | *PtRabE1b* | Potri.008G051700 | Chr08: 3058448-3063691 (-) | 651 / 216 | 23.9 / 7.65 |
|  |  | *PtRabE1c* | Potri.009G027900 | Chr09: 3873124-3876865 (-) | 648 / 215 | 23.7 / 8.37 |
|  |  | *PtRabE1d* | Potri.010G208900 | Chr10: 19838871-19843893 (+) | 651 / 216 | 24 / 7.66 |
| **RabF** | **F1** | *PtRabF1a* | Potri.008G035800 | Chr08: 1985348-1989437 (+) | 609 / 202 | 22 / 6.6 |
|  |  | *PtRabF1b* | Potri.010G226300 | Chr10: 20970320-20974110 (-) | 606 / 201 | 21.9 / 6.74 |
|  | **F2** | *PtRabF2a** | Potri.003G054900 | Chr03: 8151423-8154553 (-) | 636 / 211 | 24.1 / 7.03 |
|  |  | *PtRabF2b* | Potri.012G117800 | Chr12: 13839221-13843786 (+) | 603 / 200 | 21.8 / 6.59 |
|  |  | *PtRabF2c* | Potri.015G113000 | Chr15: 12837111-12841208 (+) | 603 / 200 | 21.7 / 5.9 |
|  |  | *PtRabF2d* | Potri.018G079300 | Chr18: 10503675-10512231 (+) | 600 / 199 | 22.6 / 9.63 |
| **RabG** | **G3** | *PtRabG3a* | Potri.001G182900 | Chr01: 15951405-15954075 (-) | 624 / 207 | 23.1 / 5.53 |
|  |  | *PtRabG3b* | Potri.002G062400 | Chr02: 4213796-4218336 (+) | 618 / 205 | 23 / 5.45 |
|  |  | *PtRabG3c* | Potri.003G053400 | Chr03: 7922177-7924769 (+) | 624 / 207 | 23.1 / 5.2 |
|  |  | *PtRabG3d* | Potri.004G153400 | Chr04: 17500350-17503062 (-) | 621 / 206 | 23 / 5.21 |
|  |  | *PtRabG3e* | Potri.005G085300 | Chr05: 6343063-6345742 (+) | 651 / 216 | 24.4 / 4.94 |
|  |  | *PtRabG3f* | Potri.005G198800 | Chr05: 21466432-21472175 (-) | 618 / 205 | 23 / 5.6 |
|  |  | *PtRabG3g** | Potri.007G079700 | Chr07: 10366505-10368670 (-) | 588 / 195 | 21.9 / 4.67 |
|  |  | *PtRabG3h* | Potri.009G115000 | Chr09: 9730706-9733618 (-) | 621 / 206 | 23 / 5.09 |
| **RabH** | **H1** | *PtRabH1a* | Potri.001G147900 | Chr01: 12033064-12036588 (+) | 468 / 155 | 17 / 5.44 |
|  |  | *PtRabH1b* | Potri.002G135500 | Chr02: 10095124-10098052 (-) | 627 / 208 | 23.1 / 7.68 |
|  |  | *PtRabH1c* | Potri.003G086700 | Chr03: 11418236-11422557 (-) | 627 / 208 | 23 / 7.68 |
|  |  | *PtRabH1d* | Potri.005G075300 | Chr05: 5513941-5516942 (-) | 624 / 207 | 23 / 6.38 |

* Four *PtRabs* (*A1b*, *D1b*, *F2a* and *G3g*) were not full-length in recent published *Populus* genome.
