# Supplementary figures and images for: Characterization of the Populus Rab family genes and the function of PtRabE1b in salt tolerance
Source: BMC Plant Biol. 2018 Jun 18;18:124. doi: 10.1186/s12870-018-1342-1 (PMC6006591; doi:10.1186/s12870-018-1342-1)

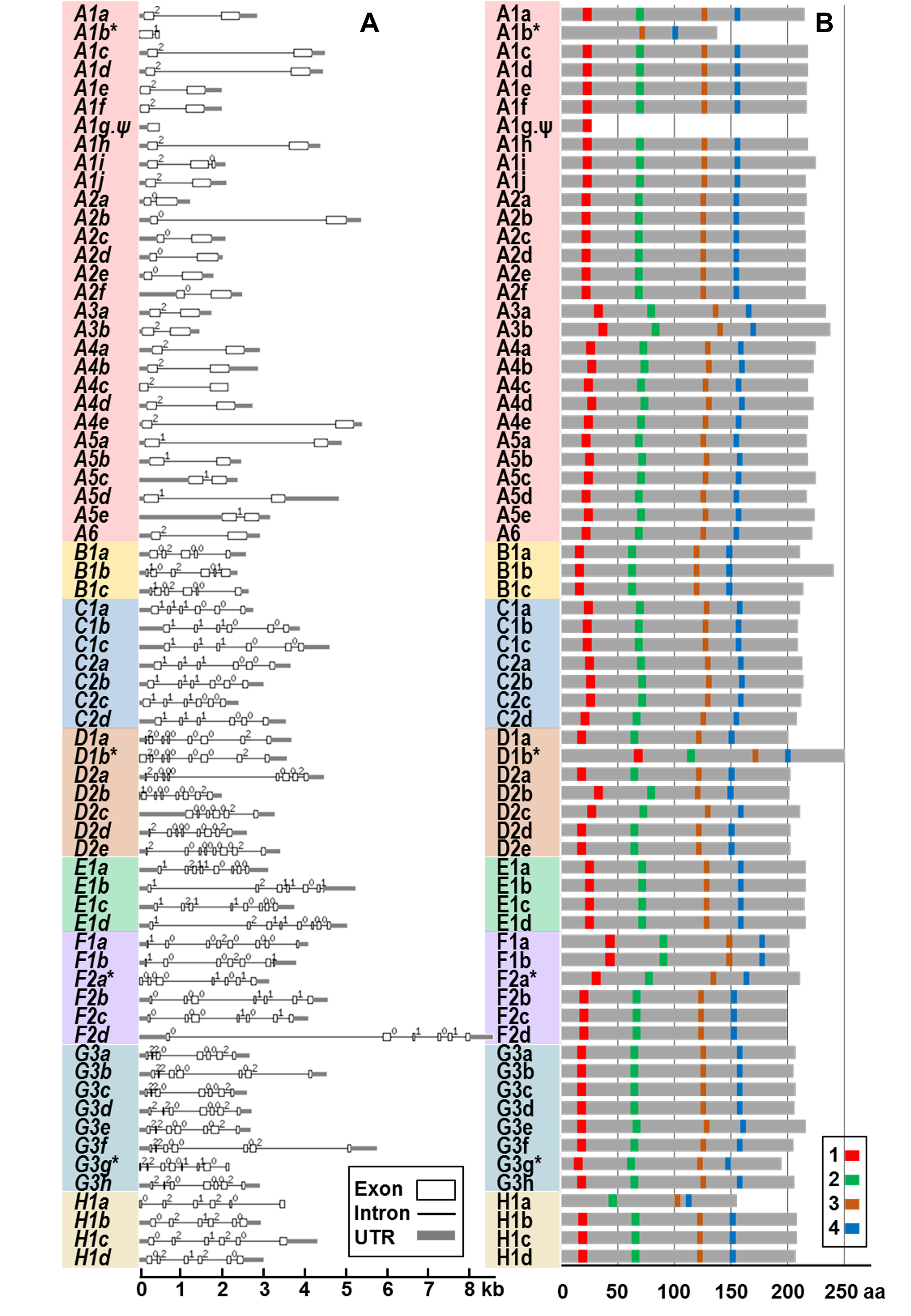

Supplement: Supplementary file 2 — Figure S1. Gene structures and motif composition of PtRabs. (A) The exon-intron structures of PtRab genes. The numbers (0, 1 and 2) indicate the splicing phases (phase 0, 1 and 2, respectively) of the Rab genes. (B) A schematic representation of conserved motifs in Rab proteins. Four conserved motifs (1–4) are represented by different colors. (TIF 3329 kb) [file 12870_2018_1342_MOESM2_ESM.tif]

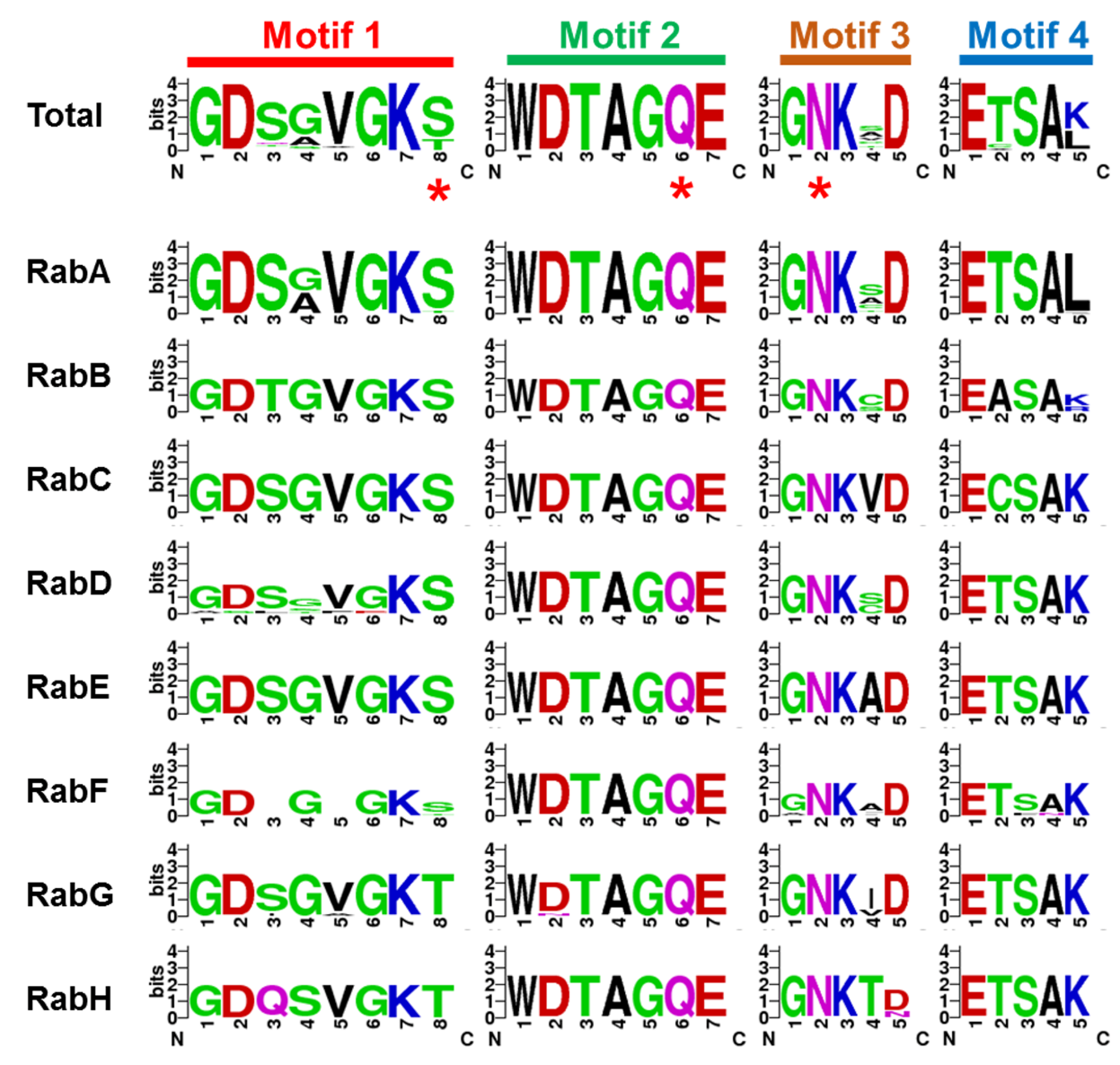

Supplement: Supplementary file 3 — Figure S2. Conserved motifs and their sequences in PtRab proteins. The four motifs are involved in nucleotide binding and hydrolysis. Mutation of the residues (shown in red asterisk) could be used to generate dominant-negative and constitutively active forms that exhibit altered nucleotide-binding or hydrolysis characteristics, which could be used to investigate Rab function. (TIF 1105 kb) [file 12870_2018_1342_MOESM3_ESM.tif]

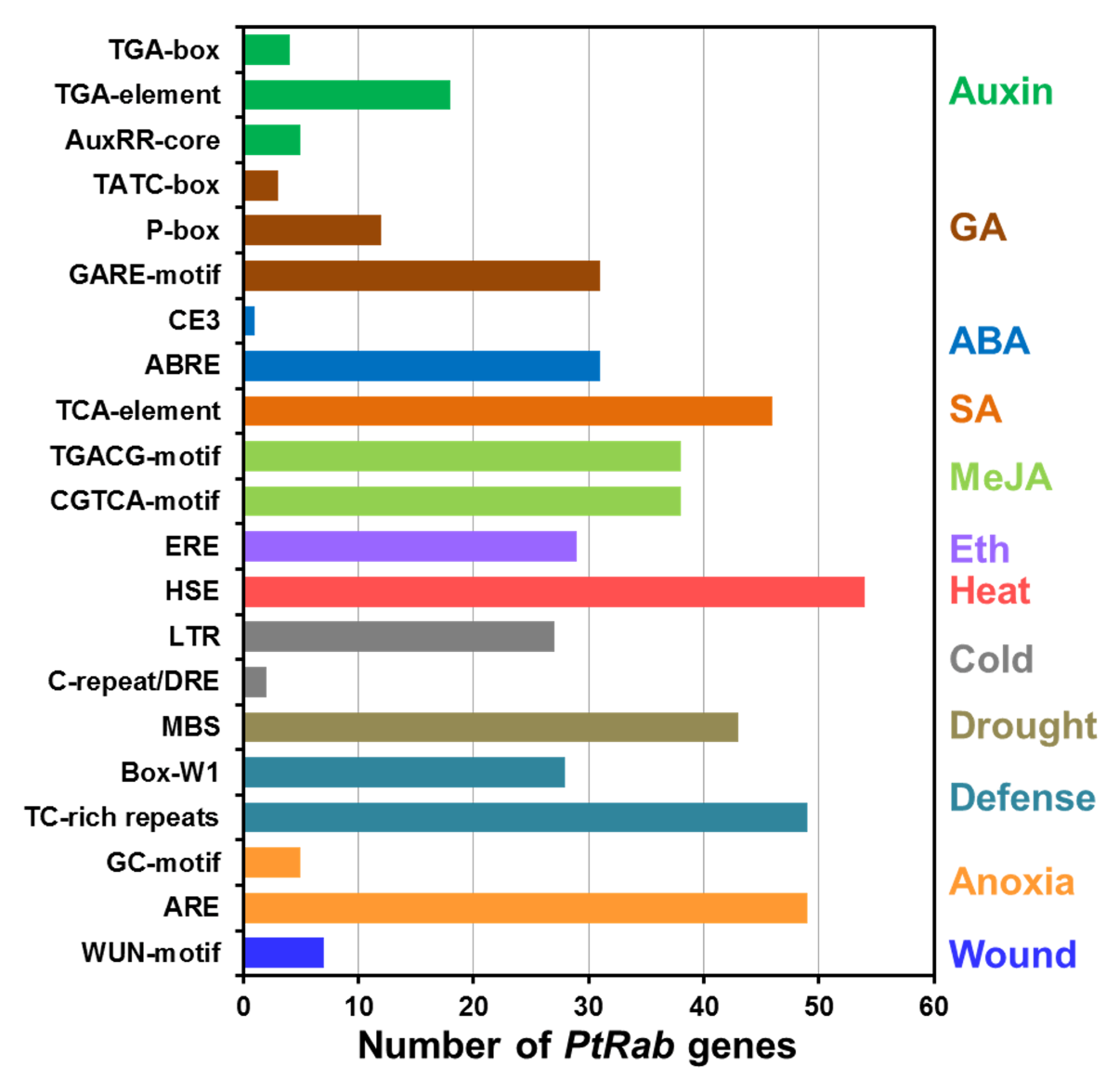

Supplement: Supplementary file 4 — Figure S3. The number of Rab genes containing various cis-acting elements. Auxin-responsive elements: TGA-element, TGA-box and AuxRR-core; gibberellin-responsive elements: TATC-box, P-box and GARE-motif; abscisic acid (ABA)-responsive elements: CE3 and ABRE; salicylic acid (SA)-responsive element: TCA-element; MeJA-responsive elements: TGACG-motif and CGTCA-motif; ethylene-responsive element: ERE; heat-responsive element: HSE; cold-responsive element: LTR and C-repeat/DRE; MYB binding site involved in drought-inducibility: MBS; defense-related elements: Box-W1 and TC-rich repeats; anoxic-related elements: GC-motif and ARE; wound-responsive element: WUN-motif. (TIF 415 kb) [file 12870_2018_1342_MOESM4_ESM.tif]

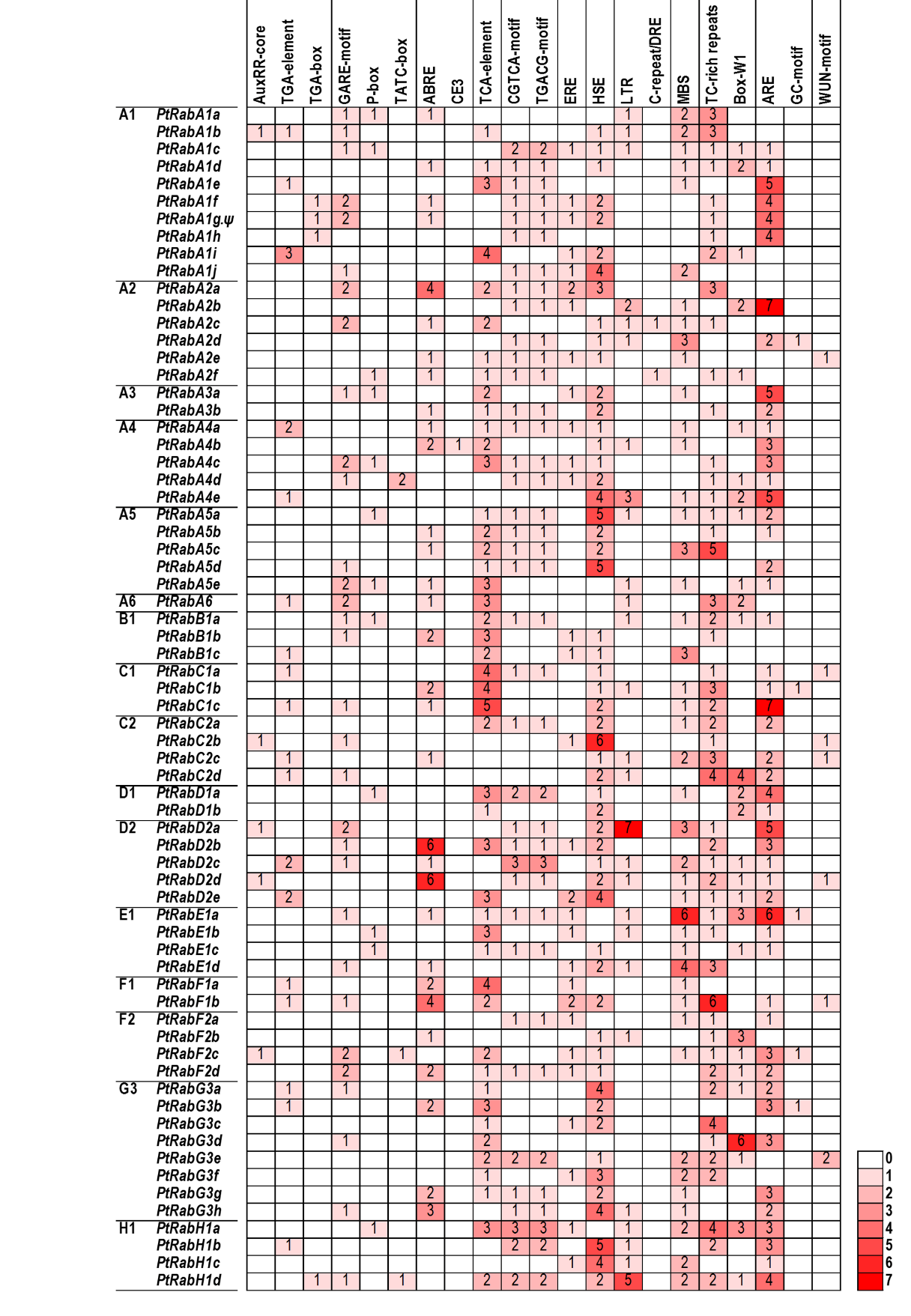

Supplement: Supplementary file 5 — Figure S4. Various cis-acting elements in responsive to hormone and stresses in Rab promoters. (TIF 1113 kb) [file 12870_2018_1342_MOESM5_ESM.tif]

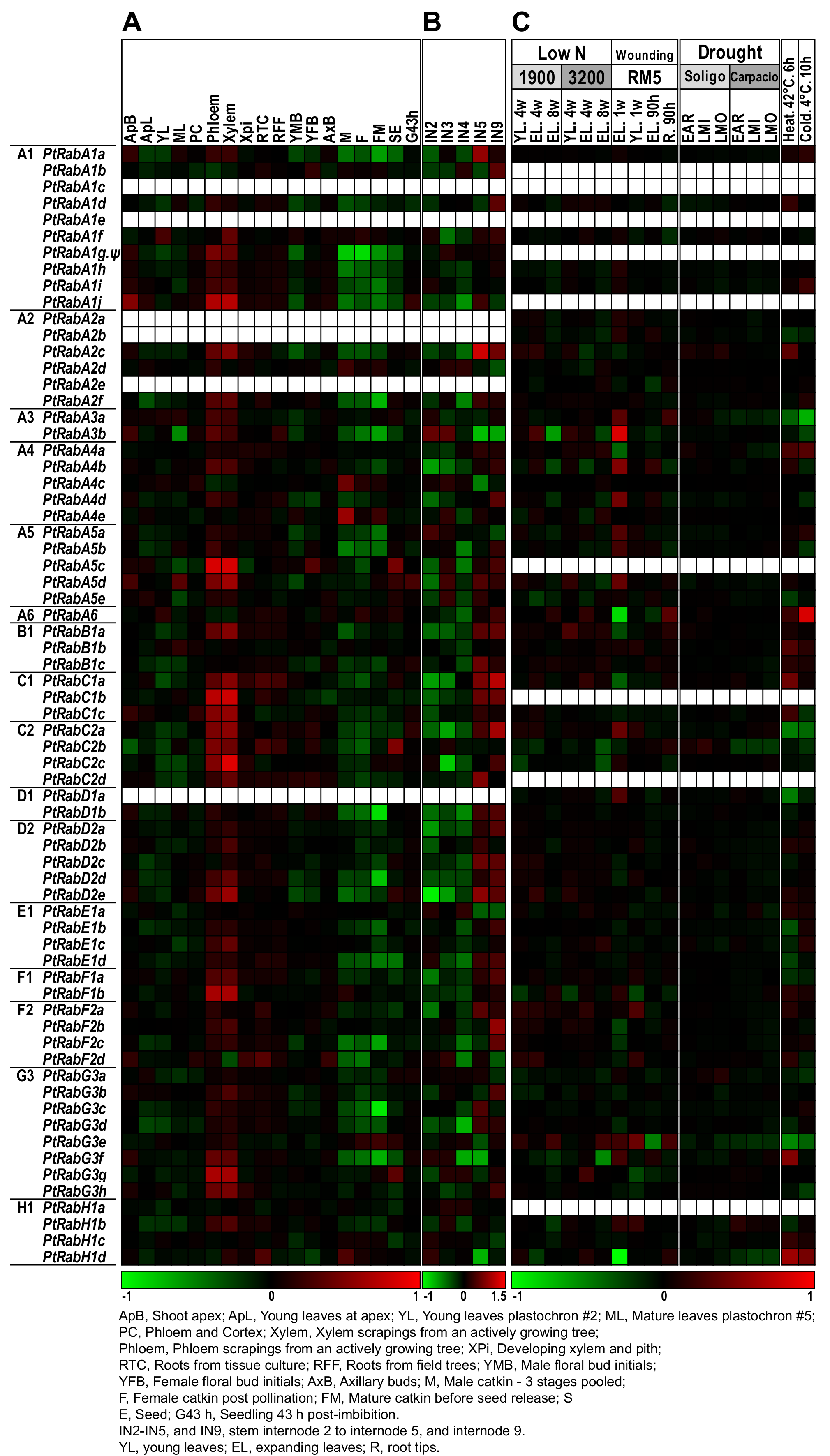

Supplement: Supplementary file 6 — Figure S5. Expression profiles of PtRab genes across different tissues and under various abiotic stresses. Heatmap showing expression of PtRab genes across tissues (A), different stem development/growth stages (B) and under various abiotic stresses (C) in poplar. For abiotic stresses, Stress treatments including: low N, nitrogen limitation; wounding, sampled either one week or 90 h after wounding; EAR, early response to water deficit by 36 h; LMI, long-term (10 day) response to mild stress with soil relative extractable water (REW) at 20–35%; LMO, long-term (10 day) response to moderate stress with soil REW at 10–20%; heat, 42 °C for 6 h; and cold, 4 °C for 10 h. Genotypes including P. fremontii × P. angustifolia clones 1979, 3200 and RM5 (low N and wounding), P. deltoids clones Soligo and Carpaccio (drought), and P. simonii (heat and cold). Color scale represents log2 expression values, green represents low level and red indicates high level of transcript abundances. Blank represents a gene has no corresponding probe sets in the microarray data. The microarray data (GSE21481, GSE21485, GSE13043, GSE16786, GSE17230, GSE41557 and GSE43872) were obtained from NCBI Gene Expression Omnibus (GEO) database. (TIF 3420 kb) [file 12870_2018_1342_MOESM6_ESM.tif]

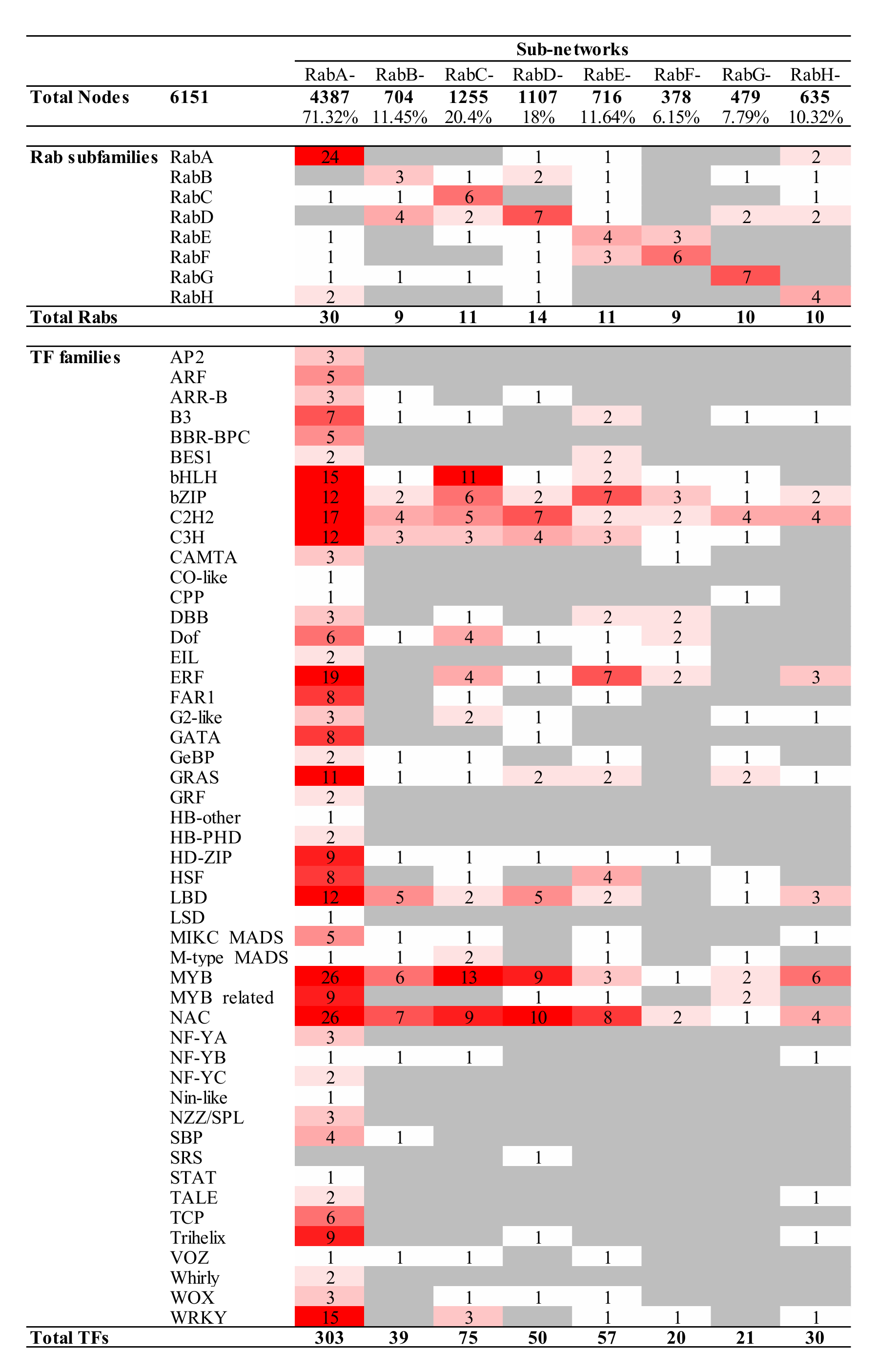

Supplement: Supplementary file 9 — Figure S6. Summary of Rab and TF subfamilies gene numbers in PtRabs co-expression sub- networks. (TIF 945 kb) [file 12870_2018_1342_MOESM9_ESM.tif]

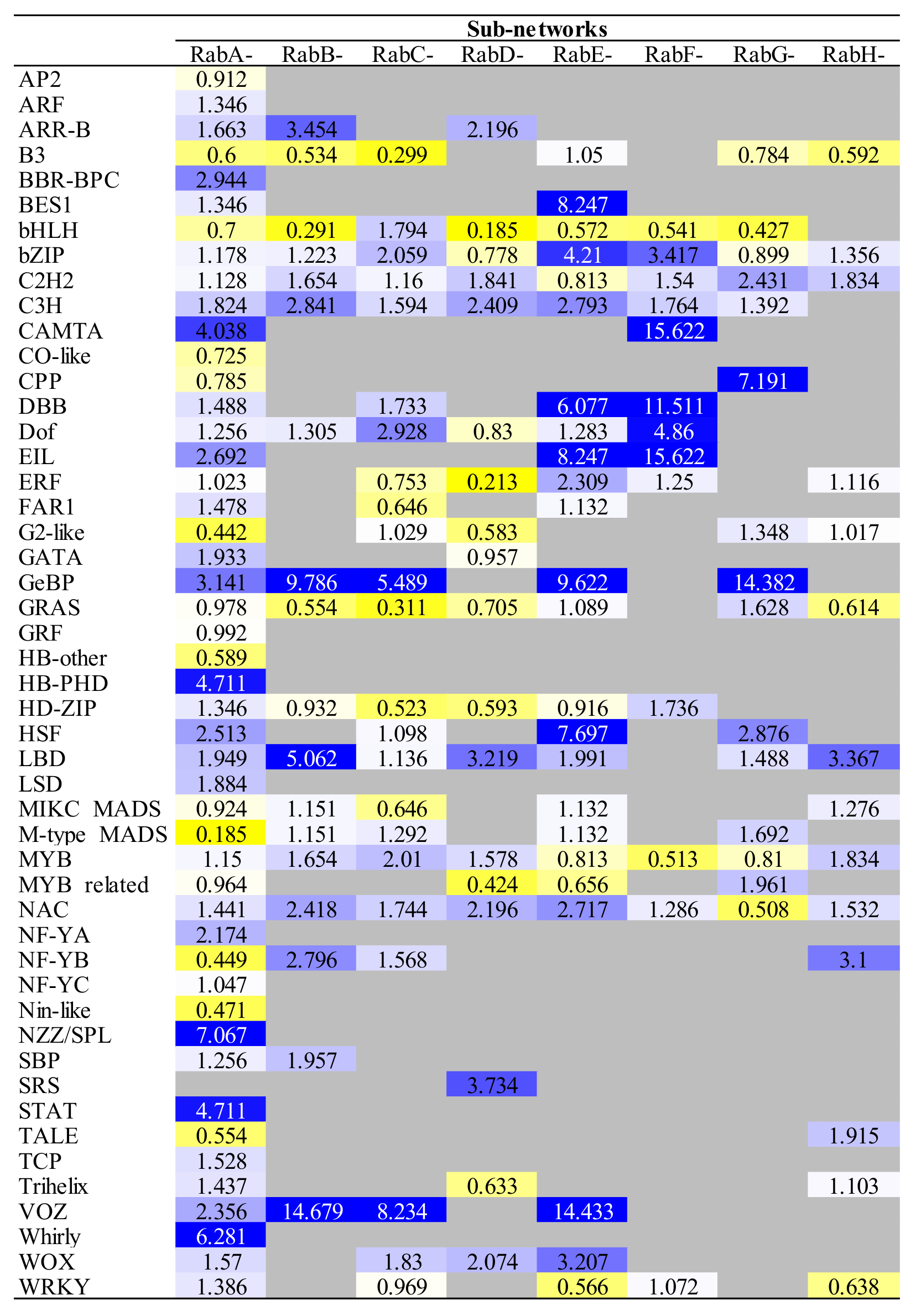

Supplement: Supplementary file 10 — Figure S7. TF enrichment of different subfamilies in PtRabs co-expression sub-networks. (TIF 1210 kb) [file 12870_2018_1342_MOESM10_ESM.tif]

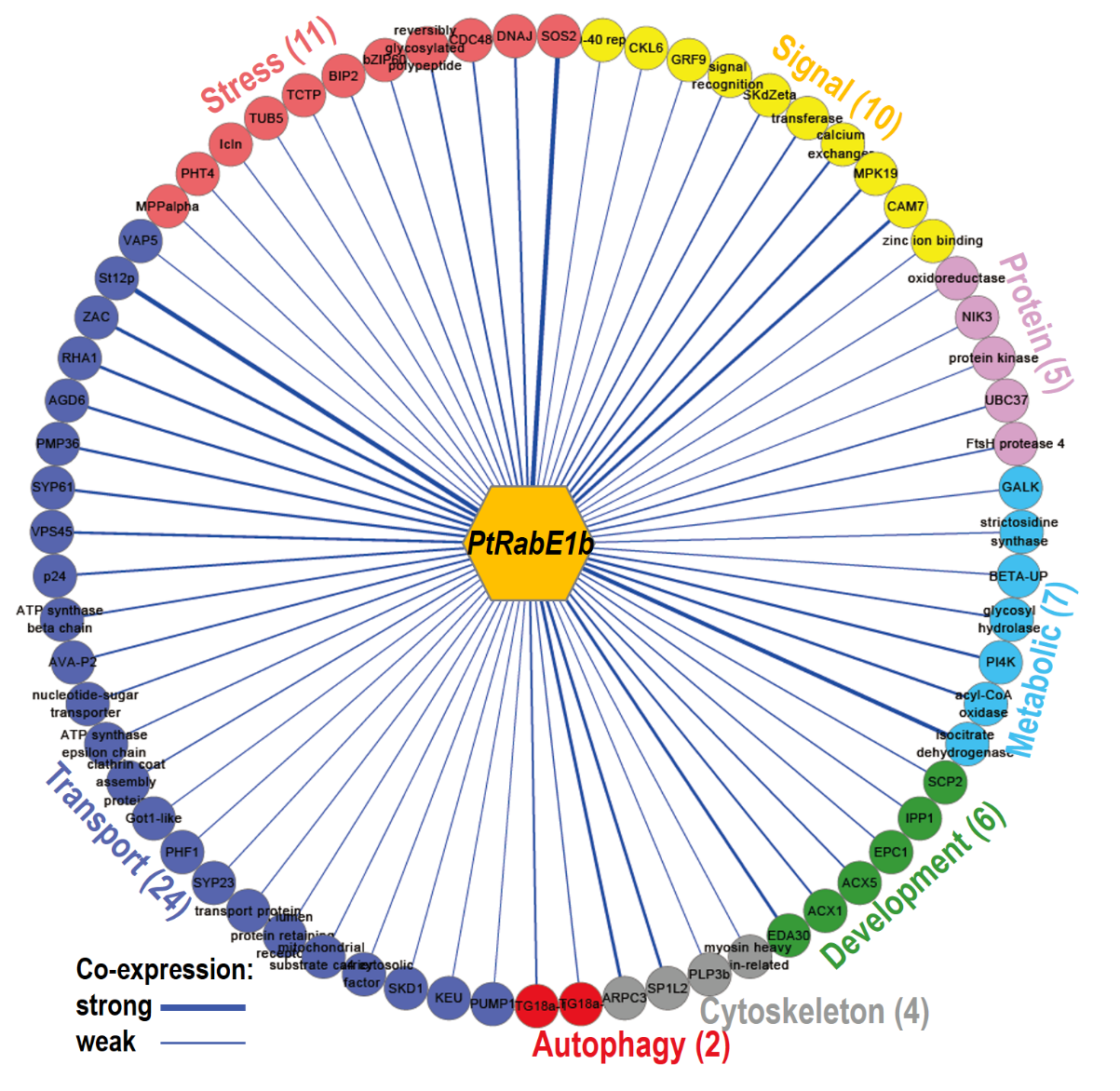

Supplement: Supplementary file 13 — Figure S8. Functional classification of genes primarily co-expressed with PtRabE1b. (TIF 1141 kb) [file 12870_2018_1342_MOESM13_ESM.tif]

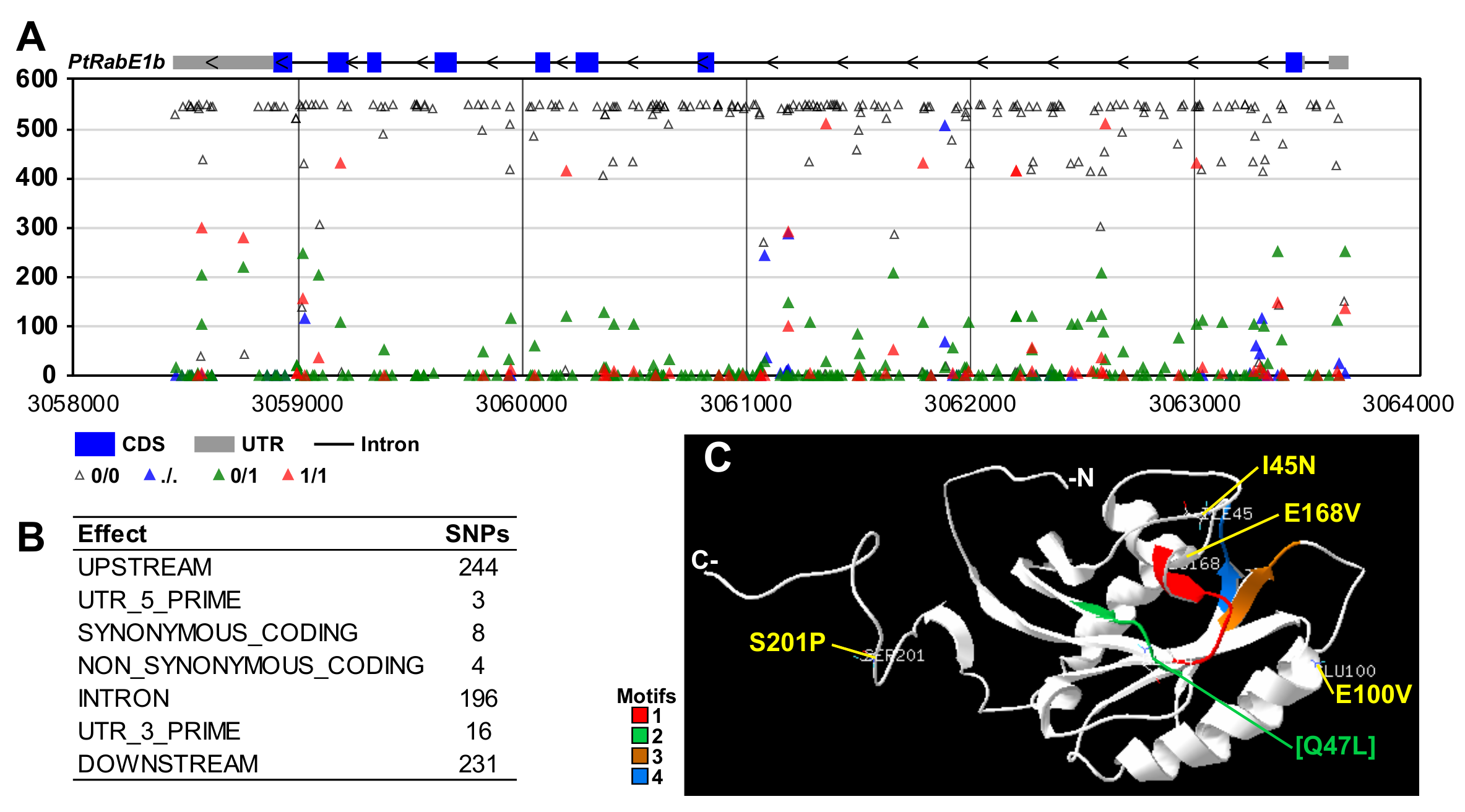

Supplement: Supplementary file 14 — Figure S9. Natural variation and protein structure of PtRabE1b. (A) Identification of SNPs in PtRabE1b from 549 P. trichocarpa individuals. (B) Effects of SNPs located in PtRabE1b. (C) Protein structure of PtRabE1b. Four non-synonymous SNPs affected proteins (I45N, E100V, E168V and S201P) were labelled as yellow. Q74L in motif-2 used for constitutive activation was labelled as green. (TIF 631 kb) [file 12870_2018_1342_MOESM14_ESM.tif]
