# Supplementary material for: Characterization of the Populus Rab family genes and the function of PtRabE1b in salt tolerance
Source: BMC Plant Biol. 2018 Jun 18;18:124. doi: 10.1186/s12870-018-1342-1 (PMC6006591; doi:10.1186/s12870-018-1342-1)
Supplement: Supplementary file 7 — Table S2. Probe sets corresponding to PtRab genes. (DOCX 22 kb) [file 12870_2018_1342_MOESM7_ESM.docx]

### Table S2. Probe sets corresponding to *PtRab* genes.

| **Subfamily** | **Gene Name** | **Gene ID** | **Corresponding Affymetrix ID** | **Corresponding NimbleGen ID** |  |  |
| --- | --- | --- | --- | --- | --- | --- |
| **A1** | *PtRabA1a* | Potri.001G374000 | Ptp.4946.1.S1_at | TREE0002S00002042 |  |  |
|  | *PtRabA1b* | Potri.004G051400 | #N/A | TREE0002S00022915 |  |  |
|  | *PtRabA1c* | Potri.004G060500 | #N/A | #N/A |  |  |
|  | *PtRabA1d* | Potri.004G061000 | PtpAffx.6112.1.S1_at | TREE0002S00020107 |  |  |
|  | *PtRabA1e* | Potri.011G060900 | #N/A | #N/A |  |  |
|  | *PtRabA1f* | Potri.011G061300 | PtpAffx.9745.1.A1_at | TREE0002S00028850 |  |  |
|  | *PtRabA1g.ψ* | Potri.011G070200 | #N/A | TREE0002S00038514 |  |  |
|  | *PtRabA1h* | Potri.011G070300 | Ptp.1415.1.S1_at | TREE0002S00001218 |  |  |
|  | *PtRabA1i* | Potri.013G123600 | PtpAffx.145773.1.S1_at | TREE0002S00038230 |  |  |
|  | *PtRabA1j* | Potri.019G092500 | #N/A | TREE0002S00012158 |  |  |
| **A2** | *PtRabA2a* | Potri.003G004100 | Ptp.6208.1.S1_s_at | #N/A |  |  |
|  | *PtRabA2b* | Potri.004G226400 | Ptp.5625.1.S1_s_at | #N/A |  |  |
|  |  |  | PtpAffx.1093.1.S1_at |  |  |  |
|  | *PtRabA2c* | Potri.006G000300 | PtpAffx.26639.2.A1_at | TREE0002S00019578 |  |  |
|  | *PtRabA2d* | Potri.008G061300 | PtpAffx.207732.1.S1_at | TREE0002S00015066 |  |  |
|  | *PtRabA2e* | Potri.010G197200 | PtpAffx.209315.1.S1_at | #N/A |  |  |
|  | *PtRabA2f* | Potri.016G000400 | Ptp.1462.3.S1_s_at | TREE0002S00028337 |  |  |
| **A3** | *PtRabA3a* | Potri.002G175700 | PtpAffx.117432.1.S1_at | TREE0002S00005934 |  |  |
|  | *PtRabA3b* | Potri.014G102200 | Ptp.612.1.S1_s_at | TREE0002S00026673 |  |  |
|  |  |  | PtpAffx.134524.1.A1_at |  |  |  |
| **A4** | *PtRabA4a* | Potri.001G270100 | Ptp.7187.1.S1_at | TREE0002S00000045 |  |  |
|  | *PtRabA4b* | Potri.005G073000 | PtpAffx.24385.1.A1_at | TREE0002S00019659 |  |  |
|  | *PtRabA4c* | Potri.006G057700 | PtpAffx.216780.1.S1_at | TREE0002S00035345 |  |  |
|  | *PtRabA4d* | Potri.007G096000 | Ptp.6386.1.S1_at | TREE0002S00004346 |  |  |
|  | *PtRabA4e* | Potri.016G050400 | PtpAffx.213313.1.S1_at | TREE0002S00016663 |  |  |
| **A5** | *PtRabA5a* | Potri.002G231800 | PtpAffx.977.1.S1_at | TREE0002S00019878 |  |  |
|  | *PtRabA5b* | Potri.002G249500 | Ptp.6418.2.S1_a_at | TREE0002S00022218 |  |  |
|  | *PtRabA5c* | Potri.006G015400 | #N/A | TREE0002S00004266 |  |  |
|  | *PtRabA5d* | Potri.014G150300 | Ptp.1488.1.S1_a_at | TREE0002S00003482 |  |  |
|  |  |  | PtpAffx.84358.1.A1_at |  |  |  |
|  | *PtRabA5e* | Potri.016G010300 | PtpAffx.65.1.S1_at | TREE0002S00001856 |  |  |
| **A6** | *PtRabA6* | Potri.015G039700 | PtpAffx.95018.1.A1_s_at | TREE0002S00004735 |  |  |
| **B1** | *PtRabB1a* | Potri.006G001500 | Ptp.522.1.S1_at | TREE0002S00002720 |  |  |
|  | *PtRabB1b* | Potri.009G159600 | PtpAffx.73678.1.S1_at | TREE0002S00018091 |  |  |
| **C1** | *PtRabB1c* | Potri.016G002200 | PtpAffx.8685.3.S1_a_at | TREE0002S00028764 |  |  |
|  | *PtRabC1a* | Potri.002G074400 | PtpAffx.27233.1.S1_at | TREE0002S00012941 |  |  |
|  | *PtRabC1b* | Potri.005G063500 | #N/A | TREE0002S00002600 |  |  |
|  | *PtRabC1c* | Potri.007G105500 | PtpAffx.249.321.S1_at | TREE0002S00004343 |  |  |
| **C2** | *PtRabC2a* | Potri.006G121400 | Ptp.4475.1.S1_s_at | TREE0002S00040514 |  |  |
|  |  |  | PtpAffx.1175.1.A1_at |  |  |  |
|  | *PtRabC2b* | Potri.008G032000 | PtpAffx.207596.1.S1_at | TREE0002S00000943 |  |  |
|  | *PtRabC2c* | Potri.010G229600 | Ptp.770.1.A1_s_at | TREE0002S00025325 |  |  |
|  |  |  | PtpAffx.88920.1.A1_at |  |  |  |
|  | *PtRabC2d* | Potri.016G097800 | #N/A | TREE0002S00004778 |  |  |
| **D1** | *PtRabD1a* | Potri.003G004000 | Ptp.1437.1.S1_s_at | #N/A |  |  |
|  | *PtRabD1b* | Potri.004G226600 | PtpAffx.30207.1.S1_s_at | TREE0002S00028637 |  |  |
|  |  |  | PtpAffx.33230.2.S1_at |  |  |  |
| **D2** | *PtRabD2a* | Potri.001G080400 | Ptp.5782.1.S1_at | TREE0002S00000155 |  |  |
|  | *PtRabD2b* | Potri.001G152800 | PtpAffx.4110.1.S1_a_at | TREE0002S00037900 |  |  |
|  | *PtRabD2c* | Potri.002G138400 | PtpAffx.4217.1.A1_s_at | TREE0002S00003967 |  |  |
|  | *PtRabD2d* | Potri.003G081800 | Ptp.5357.1.S1_at | TREE0002S00004040 |  |  |
|  | *PtRabD2e* | Potri.014G049400 | PtpAffx.4217.2.S1_s_at | TREE0002S00029573 |  |  |
| **E1** | *PtRabE1a* | Potri.001G236100 | Ptp.4604.1.S1_s_at | TREE0002S00003856 |  |  |
|  |  |  | PtpAffx.14585.3.S1_at |  |  |  |
|  | *PtRabE1b* | Potri.008G051700 | Ptp.2072.2.S1_s_at | TREE0002S00020191 |  |  |
|  |  |  | PtpAffx.12284.1.A1_s_at | | | |
|  |  |  | PtpAffx.207693.1.S1_at |  | |  |
|  | *PtRabE1c* | Potri.009G027900 | PtpAffx.14585.2.S1_a_at | TREE0002S00000617 | |  |
|  | *PtRabE1d* | Potri.010G208900 | Ptp.2072.1.S1_at | TREE0002S00003235 | |  |
|  |  |  | PtpAffx.3497.1.A1_at |  | |  |
| **F1** | *PtRabF1a* | Potri.008G035800 | Ptp.5756.1.S1_a_at | TREE0002S00000947 | |  |
|  | *PtRabF1b* | Potri.010G226300 | Ptp.5756.2.S1_at | TREE0002S00019526 | |  |
|  |  |  | PtpAffx.93137.1.S1_at |  | |  |
| **F2** | *PtRabF2a* | Potri.003G054900 | PtpAffx.113944.1.A1_at | TREE0002S00004025 | |  |
|  | *PtRabF2b* | Potri.012G117800 | Ptp.1287.1.S1_at | TREE0002S00029211 | |  |
|  |  |  | PtpAffx.99107.1.S1_s_at | | | |
|  |  |  | PtpAffx.99107.3.A1_s_at | | | |
|  | *PtRabF2c* | Potri.015G113000 | Ptp.1287.2.S1_s_at | TREE0002S00018567 | |  |
|  |  |  | Ptp.5102.1.A1_at |  | |  |
|  |  |  | Ptp.5102.1.A1_x_at |  | |  |
|  | *PtRabF2d* | Potri.018G079300 | PtpAffx.112256.1.S1_at | TREE0002S00028064 | |  |
| **G3** | *PtRabG3a* | Potri.001G182900 | Ptp.5063.1.S1_at | TREE0002S00001945 | |  |
|  |  |  | Ptp.5063.1.S1_s_at |  | |  |
|  |  |  | PtpAffx.25286.1.S1_at |  | |  |
|  | *PtRabG3b* | Potri.002G062400 | Ptp.266.1.S1_at | TREE0002S00000261 | |  |
|  |  |  | PtpAffx.18716.1.S1_at |  | |  |
|  |  |  | PtpAffx.18716.3.S1_a_at | | | |
|  |  |  | PtpAffx.18716.4.S1_at |  | |  |
|  | *PtRabG3c* | Potri.003G053400 | Ptp.768.1.S1_s_at | TREE0002S00001784 | |  |
|  | *PtRabG3d* | Potri.004G153400 | Ptp.5091.1.A1_at | TREE0002S00000429 | |  |
|  |  |  | PtpAffx.1482.1.S1_s_at |  | |  |
|  | *PtRabG3e* | Potri.005G085300 | PtpAffx.205220.1.S1_at | TREE0002S00023766 | |  |
|  | *PtRabG3f* | Potri.005G198800 | Ptp.4203.1.S1_at | TREE0002S00004226 | |  |
|  |  |  | Ptp.4203.1.S1_s_at |  | |  |
|  |  |  | PtpAffx.18716.2.A1_at |  | |  |
|  | *PtRabG3g* | Potri.007G079700 | PtpAffx.207158.1.S1_at | TREE0002S00024522 | |  |
|  | *PtRabG3h* | Potri.009G115000 | PtpAffx.1482.2.S1_a_at | TREE0002S00000544 | |  |
| **H1** | *PtRabH1a* | Potri.001G147900 | #N/A | TREE0002S00040578 | |  |
|  | *PtRabH1b* | Potri.002G135500 | PtpAffx.3422.1.S1_at | TREE0002S00002176 | |  |
|  | *PtRabH1c* | Potri.003G086700 | PtpAffx.19719.1.A1_at | TREE0002S00028697 | |  |
|  | *PtRabH1d* | Potri.005G075300 | PtpAffx.205177.1.S1_at | TREE0002S00017452 | |  |
|  |  |  | PtpAffx.219447.1.S1_s_at | | | |
