# Supplementary material for: Characterization of the Populus Rab family genes and the function of PtRabE1b in salt tolerance
Source: BMC Plant Biol. 2018 Jun 18;18:124. doi: 10.1186/s12870-018-1342-1 (PMC6006591; doi:10.1186/s12870-018-1342-1)
Supplement: Supplementary file 15 — Table S6 qRT-PCR Primers used in this study. (DOCX 18 kb) [file 12870_2018_1342_MOESM15_ESM.docx]

### Table S6. qRT-PCR Primers used in this study.

| **Gene Name** | **Gene ID** | **Forward primers** | **Reverse primers** |
| --- | --- | --- | --- |
| *PtRabA1i* | Potri.013G123600 | GCTTCGTGATCACACCGAATC | CTATCCACCTGAACATTCTTGTTGT |
| *PtRabA1j* | Potri.019G092500 | ACTTCGTGATCACACTGATTCAAAC | ATGCGGAGCAGCATCCAAC |
| *PtRabA2a* | Potri.003G004100 | AGAGCAGTAGCTACTGAGGATGCTC | CATCTTTGACACTCACAGGTGCT |
| *PtRabA2b* | Potri.004G226400 | GAAACATCTTCGAGCGGTTGC | CCGTCTTTGATACTAGCAGGTGC |
| *PtRabA3a* | Potri.002G175700 | CCATCAAGAGACTGCAGAGAGAATA | TAATATGCACTCGTCACTGCTCG |
| *PtRabA3b* | Potri.014G102200 | CCACCAAGAGAATGTGCAAGAG | ATAGTATGCGCTTGTCACTGCC |
| *PtRabB1a* | Potri.006G001500 | CCACTTGGCTAGCTGGCTTG | CCATACCCAACTTTTATCCCATAAG |
| *PtRabB1c* | Potri.016G002200 | TCACTTGGCGAGCTGGCTG | TTGCGTTCAAGTATAGCAAATGG |
| *PtRabC2b* | Potri.008G032000 | GCTCTACTCCACCAATCATGACG | ACTGCCACCACCACGAGGT |
| *PtRabC2c* | Potri.010G229600 | GCGACGGGAAACCTTCGA | GCTCCTGTGCAAGAGCCATC |
| *PtRabD2c* | Potri.002G138400 | GTAGTGTCATACGAGACAGCAAAGG | GGACAAGGCACAGAAAACTCATAA |
| *PtRabD2e* | Potri.014G049400 | GTTGTGTCATATGAGCAGGCAAA | GGCCAAAGAAAATTCATCATACTTC |
| *PtRabE1b* | Potri.008G051700 | 5' AGATCCATCAGCCAGTGGTGG | GCGACAGAAAGGGAAAAGATATG |
| *PtRabE1d* | Potri.010G208900 | 5' AACCAGATCAAGCAGCAAATGG | ACAGGAAGGGAAAAGGAGTGATATA |
| *PtRabF1a* | Potri.008G035800 | AGGGCAGGAAAGGTATGCTG | CGCATAGTCAATGCCATCTTG |
| *PtRabFb* | Potri.010G226300 | TGGACAAGAAAGGTATGCCGC | GCATATTCAATGCCATCTTGCTT |
| *PtRabG3b* | Potri.002G062400 | CTGTCCATTGCCAAAATTGCT | AGGCTAAAAACTCACAACGACCA |
| *PtRabG3f* | Potri.005G198800 | TTATCCATTGCAAAAACTGCTCTAG | ATAATGTCTCAGCATCAAGAGCTCA |
| *PtRabH1a* | Potri.001G147900 | ACCAGTCTCAGCTCTTGCAAAGTA | GTTCAGGAAAGATTGCCGACTT |
| *PtRabH1c* | Potri.003G086700 | AACTCTAACCCGACTCGACCTG | GTACTTTGCAAGAGCTGAAACTGG |
| *PtATG18a-1* | Potri.013G018400 | ACCTCTTCTGCCTGGTTGGTG | AACCCATTAGGGTTCAAAACCG |
| *PtEPC1* | Potri.008G065800 | CATAGAGTCAAAAACTAGAGGGCTG | TCCGTTTTCCCTAGCGTTTTAT |
| *PtbZIP60* | Potri.005G257900 | ATTCTCCTTCTGCTCACGAGTC | CATCAGGATCTTCCTCCTCATC |
| *PtGot1-like* | Potri.007G124400 | TTTGTGCAGAGGATACCGATTTT | GAACGTGGGCTATCTGGAGTTTA |
| *PtKEU* | Potri.001G116900 | GTGGATGAGGAGGATTCTCGC | TTTCTGTCTGAGGGAAGTGTTCAG |
| *Ptp24* | Potri.010G164600 | GCTTTCACGGCGTCGGAG | ACATTTTCTTCAGCTCCAATTCCAT |
| *PtPHF1* | Potri.010G232400 | GGTACATGCATTACATCTCTGGAGT | GCCAGGAACAGTCCTACGAGA |
| *PtSEC14* | Potri.002G243000 | TTACTCCAAGAATGCTGCTGAA | CTTTTATTTCCTGCGAAACACC |
| *PtSKD1* | Potri.004G184500 | GGTGTAGGGAACAACGATCAGAA | GCCGAGCCTTCATATCAGGTAGT |
| *PtSYP61* | Potri.004G050800 | TAGCTGGAAAAGAGTTGAATCTCAG | CAATTCTCTCCACACTTGCACTAAG |
| *PtActin* | Potri.019G006700 | ACCCTCCAATCCAGACACTG | TTGCTGACCGTATGAGCAAG |
| *PtTubulin* | Potri.001G272800 | GATTTGTCCCTCGCGCTGT | TCGGTATAATGACCCTTGGCC |
